# LncRNA PTTG3P promotes tumorigenesis and metastasis of NSCLC by binding with ILF3 to maintain mRNA stability and form a positive feedback loop with E2F1

**DOI:** 10.7150/ijbs.81738

**Published:** 2023-08-21

**Authors:** Jing Wang, Xuezhi He, Qing Yao, Chan Wang, Xiyi Lu, Rong Wang, Dengshun Miao

**Affiliations:** 1 Department of Human Anatomy, Histology and Embryology, The Research Center for Bone and Stem Cells, State Key Laboratory of Reproductive Medicine, Nanjing Medical University, Nanjing, Jiangsu, People's Republic of China.; 2 Department of Endocrinology, Changzhou Second People's Hospital Affiliated Nanjing Medical University, No.29 Xinglong Road, 213003 Changzhou, Jiangsu, People's Republic of China.; 3 Department of Oncology, The First Affiliated Hospital of Nanjing Medical University, Nanjing Medical University, Nanjing, Jiangsu, China.

**Keywords:** long non-coding RNA, PTTG3P, ILF3, non-small cell lung cancer, E2F1.

## Abstract

Non-small cell lung cancer (NSCLC) is a highly lethal disease worldwide. We found the pseudogene-derived lncRNA PTTG3P is upregulated in NSCLC and associated with larger tumor size, advanced staging, and poor prognosis. This study investigated the oncogenic roles and mechanisms of PTTG3P in NSCLC. We demonstrate that PTTG3P promoted NSCLC cell proliferation, migration, tumorigenesis, and metastasis while inhibiting apoptosis *in vitro* and *in vivo*. Mechanistically, PTTG3P formed an RNA-protein complex with ILF3 to maintain MAP2K6 and E2F1 mRNA stability, two oncogenic factors involved in NSCLC progression. RNA-seq revealed MAP2K6 and E2F1 were downregulated upon PTTG3P knockdown. RIP and RNA stability assays showed PTTG3P/ILF3 interaction stabilized MAP2K6 and E2F1 transcripts. Interestingly, E2F1 transcriptionally upregulated PTTG3P by binding its promoter, forming a positive feedback loop. Knockdown of E2F1 or PTTG3P attenuated their mutual regulatory effects on cell growth and migration. Thus, a PTTG3P/ILF3/E2F1 axis enhances oncogene expression to promote NSCLC pathogenesis. Our study reveals PTTG3P exerts oncogenic functions in NSCLC via mRNA stabilization and a feedback loop, highlighting its potential as a prognostic biomarker and therapeutic target.

## Introduction

Lung cancer is the leading cause of cancer deaths worldwide with more than 1 million deaths occurring due to lung cancer annually 1, 2. Although enormous progress has been made in lung cancer treatment, including surgery, chemotherapy, biological treatments, and immunotherapy, the overall survival is still poor mainly because patients are at advanced stages when diagnosed 3. Non-small cell lung cancer (NSCLC) accounts for approximately 85% of all lung cancer cases, and lung adenocarcinoma (LUAD) (40%) and squamous cell carcinoma (LUSC) (25-30%) are its most common histological types 4, 5. Therefore, a better understanding of the molecular mechanisms underlying the occurrence and development of NSCLC could provide a basis for improving the early diagnostic and treatment modalities, and overall prognosis in this disease.

Long non-coding RNAs (lncRNAs) are operationally defined as RNA transcripts longer than 200 nucleotides (nt) that are not translated into functional proteins. LncRNAs have recently emerged as pivotal participants in biological processes of many diseases, and are often dysregulated in a variety of cancers, including NSCLC 6-8. Emerging evidence suggests that lncRNAs play important roles in cellular development, differentiation, modulation of apoptosis, and multiple biological processes. Although there are a few well-characterized lncRNAs in NSCLC, many lncRNAs remain uncharacterized and their mechanisms of action are largely unknown 9. Pseudogene transcripts, as non-coding RNAs, were long considered to be nonfunctional relics littering the genome. However, an increasing number of studies have emphasized their significance in regulating cancer development, in particular with the implementation of the GENCODE project 10-13. Pseudogene-derived lncRNA transcripts have been extensively investigated and have been reported to be frequently dysregulated in various types of human cancer 14. Recently, the pseudogene-derived lncRNA PTTG3P (pituitary tumor-transforming 3, pseudogene) has been reported to act as an oncogene in gastric cancer 15, hepatocellular carcinoma 16, cervical cancer 17, breast cancer 18, colorectal cancer 19, and pancreatic cancer 20. These studies demonstrated that lncRNA PTTG3P was highly expressed in the above cancers, and has been linked to tumorigenesis, metastasis, cancer stage progression, and patient survival. Previous studies have demonstrated that the pseudogene-derived lncRNAs SUMO1P3 21 and DUXAP8 22 played crucial roles in NSCLC development and progression. The Zhong lab recently reported that lncRNA PTTG3P was associated with NSCLC cell proliferation, as revealed by bioinformatics analysis 23. However, the exact mechanism by which lncRNA PTTG3P regulates cancer cell proliferation and migration in NSCLC is largely unknown.

The function of lncRNAs is largely reflected by their subcellular localization. Nuclear lncRNAs mainly function in transcriptional processes, whereas cytoplasmic lncRNAs tend to function post-transcriptionally and influence signaling cascades 24, 25. There are three general mechanisms for lncRNA regulation: localization and interaction with chromatin, interaction with RNA targets, and protein modulation. A single lncRNA can function via different mechanisms, suggesting that their regulation of gene expression is complex 24. LncRNAs are known to function in gene expression through their interaction with different RNA targets 24. Interactions between mRNAs and lncRNAs can regulate RNA metabolism through the recruitment of factors involved in splicing, mRNA stability, and translation 24, 25. Subcellular location of lncRNA PTTG3P was predicted by using lncRNA subcellular localization predictor software and subcellular fractionation, which indicated that lncRNA PTTG3P was mainly localized to the cytoplasm 20. Mechanistically, lncRNA PTTG3P plays important roles in cancer initiation and progression by serving as competing endogenous RNA (ceRNA) via competitive binding to shared microRNAs (miRNAs), thus affecting both their cognate genes and unrelated genes 16, 18-20, 26. Whether lncRNA PTTG3P can regulate RNA metabolism through the recruitment of factors involved in splicing, mRNA stability, and translation remains unknown.

Extensive evidence suggests that lncRNA expression can be regulated to a certain extent, similar to protein-coding genes. For example, p53 enhances lncRNA-p21 transcription and promotes cell growth arrest and apoptosis 27. Recently, a study demonstrated that FoxM1-mediated transcription of lncRNA PTTG3P promotes tumorigenesis and metastasis of pancreatic cancer 20. However, the regulators involved in the abnormal expression of lncRNA PTTG3P in NSCLC cells remain unclear.

In the present study, we identified a highly expressed lncRNA PTTG3P in NSCLC samples by analyzing a public dataset and verified it in 60 paired NSCLC tissues and paracancerous tissues using qRT-PCR. To explore the bio-function of lncRNA PTTG3P in NSCLC, we performed loss-of- and gain-of-function experiments in NSCLC cell lines. The effects of PTTG3P on cell viability were evaluated by MTT and colony-formation assays. Cell migration and invasion were detected by Transwell assay. Cell cycle and cell apoptosis were evaluated by flow cytometry. Nude mice model was used to examine the effects of PTTG3P on tumor cell growth and metastasis *in vivo*. Proteins that interacted with lncRNA PTTG3P were identified by RNA pull-down, mass spectrometry, and RIP analyses. The common downstream targets of the PTTG3P/ interleukin enhancer-binding factor 3 (ILF3) complex were identified using RNA transcriptome sequencing, bioinformatics analysis, and qRT-PCR validation. Transcriptional regulation of lncRNA PTTG3P by E2F1 was examined using chromatin Immunoprecipitation (ChIP) and luciferase assays. The results from this study demonstrate that lncRNA PTTG3P promotes tumorigenesis and metastasis of NSCLC by binding to ILF3 to maintain MAP2K6 and E2F1 mRNA stability and form a positive feedback loop with E2F1.

## Materials and Methods

### Patients and tissue samples

A total of 60 primary NSCLC patients who had undergone surgical treatment at the First Affiliated Hospital of Nanjing Medical University, China, between 2016 and 2020 were included in this study, and 60 paired NSCLC and paracancerous tissues were harvested and stored in liquid nitrogen. None of these patients had received chemotherapy or radiotherapy prior to surgery. Tumor sizes and tumor-node-metastasis (TNM) stages were determined according to the National Comprehensive Cancer Network (NCCN) criteria. Informed consent was obtained from all participants, and the collection of tissue samples was approved by the Internal Review and Ethics Boards at the First Affiliated Hospital of Nanjing Medical University. Supplemental Table S1 summarizes the clinical characteristics of NSCLC patients.

### Cell culture

Three human NSCLC adenocarcinoma cell lines (SPCA1, A549, and PC9), three NSCLC squamous carcinomas cell lines (H1703, SK-MES-1, and H226), and one normal human bronchial epithelial cell line (16HBE) were purchased from the Institute of Biochemistry and Cell Biology of the Chinese Academy of Sciences (Shanghai, China). SPCA1, PC9, and H1703 were cultured in DMEM (GIBCO-BRL, NY, USA) medium, A549 and H226 were cultured in RPMI 1640 (GIBCO-BRL, NY, USA) medium, while SK-MES-1 was cultured in EMEM (GIBCO-BRL, NY, USA) medium. All cells were supplemented with 10% fetal bovine serum (FBS, AUS), 100 U/ml penicillin, and 100 mg/ml streptomycin and kept in an incubator at 37 °C with 5% CO_2_.

### Rapid amplification of cDNA ends (RACE)

5´-RACE, 3´-RACE, and full sequence amplification of PTTG3P were performed using the SMART RACE cDNA Amplification Kit (Takara Biotechnology [Dalian] Co., Ltd.), according to the manufacturer's instructions. The primers used in 3'-RACE and 5'-RACE are listed in the Supplemental Table S2.

### RNA extraction and quantitative reverse transcriptase polymerase chain reaction (qRT-PCR) analysis

The total RNA was extracted from frozen tissues or cultured cells using TRIzol reagent (Invitrogen, CA, USA), according to the manufacturer's instructions. For qRT-PCR, the isolated RNA was reverse transcribed to cDNA using a PrimeScript RT reagent Kit (Vazyme, Nanjing, China). Real-time PCR analyses were performed with SYBR Green (Vazyme, Nanjing, China). Results were normalized to the expression of glyceraldehyde-3-phosphate dehydrogenase (GAPDH). The gene-specific primers were synthesized by GENEray, as shown in Supplemental Table S2. The qRT-PCR and data collection were conducted using an ABI 7500 real-time PCR system (Applied Biosystems, Foster City, CA, USA). Our qRT-PCR results were analyzed, expressed relative to the threshold cycle (CT) values, and then converted to fold changes.

### Subcellular fractionation location

Separation of the nuclear and cytosolic fractions was performed by using the PARIS Kit (Cat. AM1921, Invitrogen, CA, USA), according to the manufacturer's instructions.

### Plasmid and small interfering RNA (siRNA) transfection

Cells cultured on six-well plates were transfected with siRNA or non-specific control siRNA (si-NC) using Lipofectamine 2000 (Invitrogen, Shanghai, China), according to the manufacturer's protocol. The sequences for siRNAs are listed in Supplemental Table S3. The PTTG3P plasmid was synthesized according to the sequence of RACE (based on the PTTG3P sequence, NR_002734.2, in NCBI) and then subcloned into a pCDNA3.1 vector (Invitrogen, Shanghai, China). An empty pcDNA3.1 vector was used as a control. Plasmid transfection was carried out by using X-tremeGENE™ HP DNA (Roche, Basel, Switzerland). Cells were harvested after 48 h for qRT-PCR and other experiments.

### Cell viability and cell proliferation assays

The cell viability assay was tested by using an MTT kit (Biofroxx). The A549 and H1703 cells were grown in 96-well plates (2500-3000 cells/well) and cultured for 24 h after being transfected with plasmid DNA or siRNA. MTT assay was used to detect the relative cell viability every 24 h, according to the manufacturer's instructions. For each well, 20 µl of MTT solution (5 mg/ml) was added and measured spectrophotometrically at 490 nm after 4 h of incubation. For the colony-formation assay, a certain number of transfected cells were placed in each well of the 6-well plates and maintained in proper media containing 10% FBS for 12 to 14 days, replacing the medium every 5 days. Then, the colonies were fixed with methanol and stained with 0.1% crystal violet (Sigma-Aldrich, USA) in PBS for 15 min. The colony-formation capabilities were analyzed by counting the number of stained colonies. For each group, wells were evaluated simultaneously in triplicate, and experiments were repeated three times independently.

### EdU analysis

Proliferating cells were evaluated by using a 5-ethynyl-2-deoxyuridine (EdU) labeling/detection kit (Ribobio, Guangzhou, China), according to the manufacturer's protocol. Hoechst 33342 (5 μg/ml) was used to label cell nuclei (blue), followed by observation under a fluorescence microscope at 200× magnification. The percentage of EdU-positive cells (red) was calculated from three random fields in three wells. The EdU incorporation rate was expressed as the ratio of EdU-positive cells to total Hoechst 33342-positive cells, which were counted using Image-Pro Plus (IPP) 6.0 software (Media Cybernetics). A higher percentage of positive spots represents a higher proliferation capacity of cells.

### Cell migration and invasion assays

For cell migration assay, 24-well Transwell chambers with an 8-mm pore size polycarbonate membrane were used (CORNING, NY, USA). An aliquot of 3×10^4^ cells was seeded on the top of the membrane. After incubation for 24 h, cells inside the upper chamber were removed with a cotton swab, whereas cells on the surface of the lower membrane were fixed and then stained with 0.5% crystal violet (Sigma-Aldrich, USA). For cell invasion assay, 8×10^4^ cells were seeded on the top of the membrane pre-coated with Matrigel matrix (30ug, Corning, NY, USA). After incubation for 48-72 h, cells on the surface of the lower membrane were fixed and then stained. Three randomly selected fields were counted in each well by using an inverted microscope at 100× magnification.

### Flow-cytometric analysis

For the cell apoptosis analysis, transfected cells were harvested after 36 to 48 h transfection by trypsinization without EDTA. Following that, double staining with fluorescein isothiocyanate (FITC)-Annexin V and propidium iodide (PI) was done by the FITC Annexin V Apoptosis Detection Kit (BD Biosciences, NY, USA), according to the manufacturer's instructions. The cells were analyzed with a flow cytometer (FACScan; BD Biosciences) equipped with Cell Quest software (BD Biosciences, NY, USA). Cells were categorized as viable cells, dead cells, early apoptotic cells, and late apoptotic cells, and then the relative ratio of apoptotic cells that was displayed was compared with control transfection. For the cell-cycle analysis, the cells were stained with PI by using the CycleTEST^TM^ PLUS DNA Reagent Kit (BD Biosciences), following the manufacturer's protocol, and analyzed by FACScan. The percentages of cells in the G0/G1, S, and G2/M phases were counted and compared. All samples were assayed in triplicate.

### Tumor formation and tumor metastasis in nude mice

Four-week-old male athymic BALB/c nude mice were maintained under specific pathogen-free conditions and manipulated according to protocols approved by the Shanghai Medical Experimental Animal Care Commission. A549 cells which stably transfected with LV-shPTTG3P or LV-shCTR (empty vector) or pcDNA-PTTG3P were collected and harvested at a concentration of 2×10^7^ cells/ml with physiological saline. Lentivirus carrying shPTTG3P and empty vector (negative control) was purchased from GENECHEM (Shanghai, China). ShPTTG3P that stably expressed A549 cells were established by infecting with lentivirus and selected by puromycin (Med Chem Express, USA). For the tumorigenicity studies, cells were subcutaneously injected into a single side of the subcutaneous armpit of each mouse (2×10^6^). Tumor sizes were examined every 3 days in mice. Tumor volumes were measured as length×width^2^ ×0.5. Twenty-one days after injection, the tumors were removed from all the animals, and tumor weights were taken. The primary tumors were excised for further analysis. Tumor tissues were used to perform qRT-PCR analysis of PTTG3P levels and immunostaining analysis of Ki-67 and Bcl-2 protein expression. Images of Hematoxylin-Eosin (HE) staining and immunostaining analysis were taken by using a microscope at 200× magnification. For the tumor metastasis studies, an aliquot of 2×10^6^ stably-transfected A549 cells were collected and injected into the tail vein of each mouse. The mice were euthanized after 8 weeks, and the lung tissues were removed; the tumors on the lung tissues were counted, and photos were taken and recorded at the same time. For lung tissues, pictures of HE staining were taken by using a microscope at 50× magnification. The protocol was approved by the Committee on the Ethics of Animal Experiments of the Nanjing Medical University. All of the experimental procedures involving animals were carried out in strict accordance with the recommendations in the Guide for the Care and Use of Laboratory Animals of the National Institutes of Health.

### Western blot and antibodies

Cell protein lysates were separated by 10% sodium dodecyl sulfate-polyacrylamide gel electrophoresis (SDS-PAGE), transferred to 0.22 μm polyvinylidene fluoride (PVDF) membranes (Millipore, USA), and incubated with specific antibodies. Autoradiograms were quantified by densitometry (Quantity One software; Bio-Rad, CA, USA). GAPDH antibody was used as a control. Anti-E2F1 (ab179445, 1:1000), -CDK4 (ab199728, 1:1000), -CDK6 (ab199728, 1:1000), -Ki67 (ab16667, 1:1000), -Bcl-2 (ab182858, 1:1000), -ILF3 (ab131004, 1:1000), -MAP2K6 (ab33866, 1:1000), and -GAPDH (ab9485, 1:2000) antibodies were purchased from Abcam. Anti -CDK6 (3136s, 1:1000), -P21 (2947s, 1:1000) were purchased from Cell Signaling Technology.

### *In vitro* transcription assays and RNA pull-down mass spectrometry (LC-MS/MS) assays

The *in vitro* translation assays were performed using an mMACHINE™ T7 Transcription Kit, according to the manufacturer's instructions (Cat. AM1344, Invitrogen, CA, USA). Then, PTTG3P RNAs were labeled by desthiobiotinylation with a Pierce RNA 3´ End Desthiobiotinylation Kit (Cat. 20164, Magnetic RNA-Protein Pull-Down Kit, Components, Thermo, USA). Then, RNA pulldown assays were performed using a Pierce Magnetic RNA-Protein Pull-Down Kit, according to the manufacturer's instructions (Cat. 20164, Magnetic RNA-Protein Pull-Down Kit, Thermo, USA). After elution of the lncRNA-interacting proteins, the samples were subjected to a mass spectrometric analysis. The LC-MS/MS experiments were performed with an LTQ linear trap mass spectrometer (Thermo Finnigan, San Jose, CA, USA) equipped with a micro spray source, which was provided by HOOGEN BIOTECH (Shanghai, China). A list of the top ten potential PTTG3P-interacting protein candidates in A549 cells based on MS analysis was shown in Supplemental Table S4.

### RNA immunoprecipitation (RIP) assays

RIP experiments were performed using a Magna RIP™ RNA-Binding Protein Immunoprecipitation Kit (Cat. 17-701, Millipore, USA), according to the manufacturer's instructions. The antibodies for RIP assays of ILF3 were obtained from Abcam.

### Chromatin Immunoprecipitation (ChIP) assays

A549 cells were treated with formaldehyde and incubated for 10 min to generate DNA-protein crosslinks. Cell lysates were then sonicated to generate chromatin fragments of 200-300 bp and immunoprecipitated with E2F1-specific antibody (Cell Signaling Technology) or IgG as a control. Precipitated chromatin DNA was recovered and analyzed by qPCR and the amplified DNA was analyzed by agarose (2%) gel electrophoresis for visualization. The ChIP assays were performed using a Magna ChIP™ Protein A Magnetic Beads (Cat. 17-610, Millipore, USA), according to the manufacturer's instructions. The promotor primers of PTTG3P used for the analysis of E2F1 binding are listed in Supplemental Table S2.

### Dual-luciferase reporter assay

The specific DNA fragments containing the wild type or mutant PTTG3P fragment were sub-cloned downstream of the luciferase gene within the pGL3-Baisc luciferase reporter vector (GENECHEM Co., Ltd., Shanghai, China). Human A549 cells (1.0×10^5^) grown in a 24-well plate were co-transfected with 500 ng of either empty vector or E2F1 construct; 500 ng of firefly luciferase reporter comprising wild type or mutant PTTG3P using X-tremeGENE™ HP DNA (Roche, Shanghai, China) was used. Forty-eight hours after transfection, luciferase assay was performed by using the Dual-Luciferase Kit (Promega, USA). The relative firefly luciferase activities were normalized to those of Renilla luciferase. All samples were assayed in triplicate.

### RNA transcriptome sequencing

The total RNA from 1×10^6^ cells was isolated using TRIzol reagent and quantified. The concentration of each sample was measured with a NanoDrop 2000 (Thermo Scientific, USA). The quality was assessed by an Agilent 2200 (Agilent, USA). The sequencing library of each RNA sample was prepared using an Ion Proton Total RNA-Seq Kit v2, according to the protocol provided by the manufacturer (Life Technologies, USA). Supplemental Table S5 showed mRNA variation abundance (>1.5-fold) in PTTG3P-knockdown A549 cells depending on RNA-Seq analysis.

### Gene Ontology (GO) analysis

A GO analysis was performed to facilitate the elucidation of the biological implications of unique genes in the significant or representative profiles of the target genes of the differentially expressed mRNAs in the experiment. We downloaded the GO annotations from NCBI (https://www.ncbi.nlm.nih.gov/), UniProt (https://www.uniprot.org/), and Gene Ontology Resource (http://geneontology.org/). Fisher's exact test was applied to identify the significant GO categories, and FDR was used to correct the p-values.

### mRNA stability analysis

A549 and H1703 cells, which transfected siRNAs against lncRNA PTTG3P or ILF3, were seeded into 6-well plates to get 50% confluency after 24 h. Cells were treated with 5 μg/ml actinomycin D (Med Chem Express, USA) and collected at indicated time points. Total RNA was extracted and the mRNA expression levels were analyzed by RT-PCR. To measure the half-life of endogenous MAP2K6 and E2F1 mRNA, actinomycin D was added into the medium and total RNA was prepared at the times indicated and subjected to qRT-PCR analysis using specific primers. mRNA levels were normalized to 18 S rRNA and plotted as a percentage of the value at time zero (set at 100%).

### Statistical analysis

All statistical analyses were performed using SPSS 22.0 software (IBM, SPSS, USA). The significance of differences between groups was estimated by Student's t-test and chi-square test, as appropriate. Two-sided p values were calculated, and a probability level of 0.05 was considered to be statistically significant.

## Results

### PTTG3P is highly expressed in human NSCLC tissues and positively correlated with tumor size and advanced TNM stage

**Figure 1:**
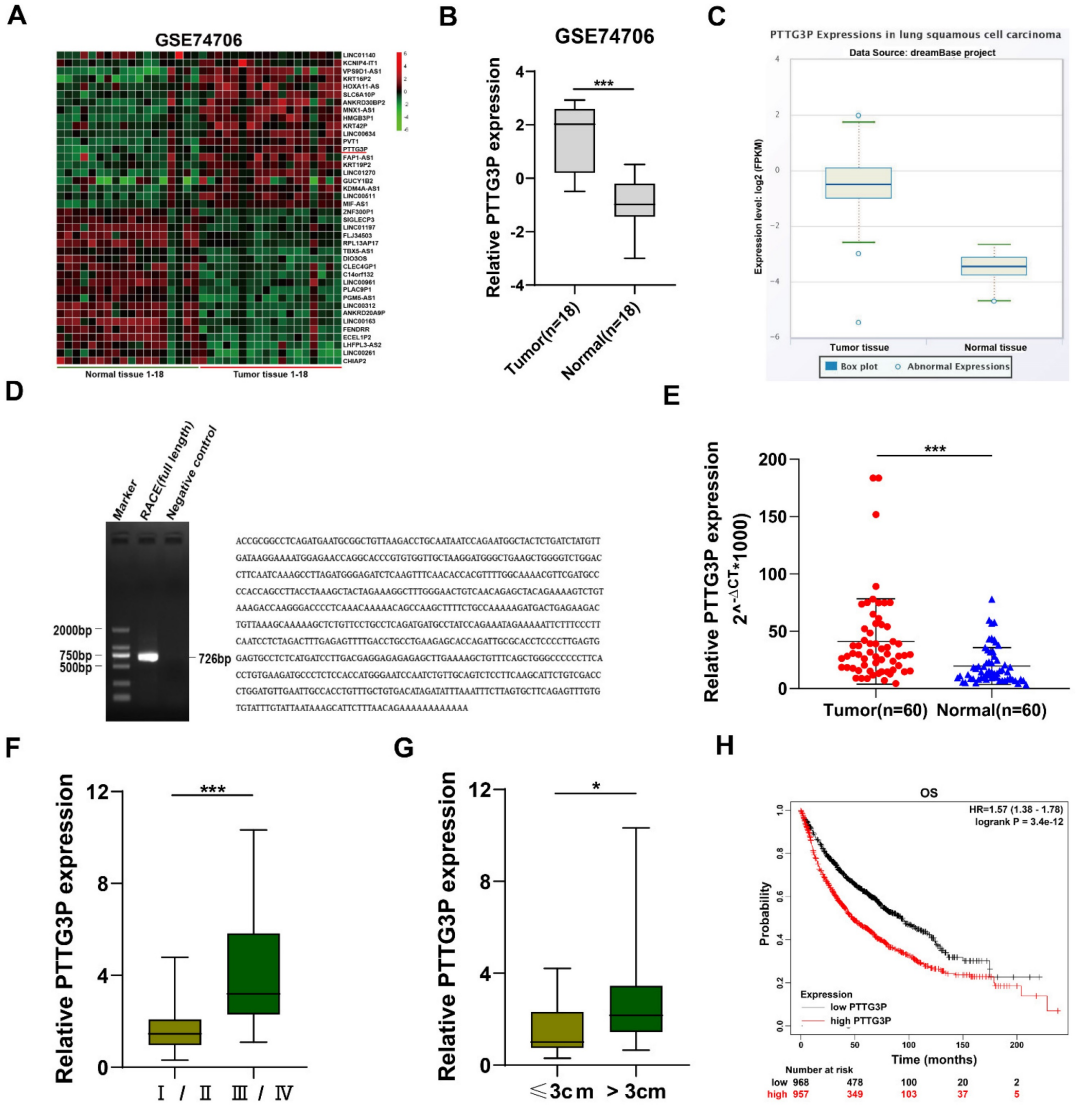
** Relative PTTG3P expression in NSCLC tissues and its clinical significance. A.** Raw microarray data were downloaded from GSE74706 data (data from GEO Database). **B.** Relative expression of PTTG3P in human lung cancer tissues (n=18) and corresponding adjacent tissues (n=18) in GSE74706 data. **C.** PTTG3P expression levels were analyzed in human lung squamous cell carcinoma tissues and normal tissues from database dreamBase (http://rna.sysu.edu.cn/dreamBase/). **D.** The full sequence of PTTG3P was confirmed by RACE. **E.** Relative PTTG3P expression in human NSCLC tissues (n=60) compared with corresponding paracancerous tissues (n=60) was examined by qRT-PCR and normalized to GAPDH expression. The △Ct value was determined by subtracting the GAPDH Ct value from the PTTG3P Ct value (relative to a single reference value). **F, G.** Analysis of relationship between PTTG3P expression and pathological stages (I/II or IIII/IV) and the tumor size (>3 cm or ≤3 cm) in 60 patients with NSCLC. **H.** Kaplan-Meier overall survival curves were analyzed according to online survival analysis software Kmplot (http://www.kmplot.com/lung/). *: p<0.05, ***: p<0.001.

To identify the dysregulated lncRNAs in NSCLC tissues, we downloaded the data of NSCLC RNA expression profile from the GEO, GSE74706 data set (https://www.ncbi.nlm.nih.gov/gds). The top 20 upregulated or downregulated lncRNAs in NSCLC are shown in Fig. 1A. Since lncRNAs with high expression are more feasible for use as markers for early diagnosis or response to treatment of NSCLC, we chose lncRNA PTTG3P, which is a lncRNA highly expressed in NSCLC. LncRNA PTTG3P expression level in NSCLC was 2.51-fold higher than that in normal lung tissue on statistical analysis of PTTG3P expression level in NSCLC using 18 pairs from GSE74706 datasets (Fig. 1B). To further verify the results of the GEO database, we analyzed the expression level of lncRNA PTTG3P in LUSC in RNA sequence data of the dreamBase project (http://rna.sysu.edu.cn/dreambase/). The results showed that lncRNA PTTG3P expression was also significantly upregulated in LUSC (Fig. 1C). To further study the function of lncRNA PTTG3P in NSCLC, we first determined the full-length sequence of PTTG3P in A549 cells, based on the sequence information provided by NCBI, and we used RACE technology to amplify and determine its sequence. Compared with the sequence archived in the RefSeq database, we determined the full-length sequence of PTTG3P with increased 89 bp in 3´RACE and 91 bp in 5´RACE (Fig. 1D). The expression level of PTTG3P was examined by using qRT-PCR in 60 paired NSCLC and paracancerous tissues harvested by us. The results showed that the expression level of lncRNA PTTG3P in NSCLC tissues was 2.16 times higher than that in adjacent tissues (Fig. 1E). According to the median expression of lncRNA PTTG3P, 60 samples were divided into two groups: PTTG3P high expression group (n=30) and PTTG3P low expression group (n=30). Pearson's Chi-square test (Fisher's exact test was used when the expected count of variable was less than 5) was used to test the correlation between PTTG3P expression level and clinicopathological factors. The results showed that PTTG3P level was positively correlated with advanced tumor-node-metastasis (TNM) stage (*p*=0.001) and tumor size (*p*=0.017). However, there was no statistically significant relationship between PTTG3P expression and other clinical factors, such as gender (*p*=1), tumor grade (*p*=0.083), or smoking history (*p*=0.573) (Supplemental Table S1). PTTG3P expression level was significantly higher in stage III/IV patients than in stage I/II patients, and in patients with tumor size greater than 3 cm than in patients with tumor size less than 3 cm (Fig. 1F and G). In addition, we analyzed the data of 1,926 NSCLC patients from open data (http://www.kmplot.com/lung/) and used Kaplan Meier plotter to study the correlation between PTTG3P expression level and survival rate 28. The results showed that the higher the lncRNA PTTG3P expression level, the worse the overall survival rate (OS) (Fig. 1H). These results indicate that lncRNA PTTG3P is highly expressed in NSCLC, and its expression level is related to NSCLC cell growth, metastasis, and prognosis.

### 3.2 PTTG3P stimulates NSCLC cell proliferation and inhibits their apoptosis *in vitro*

**Figure 2:**
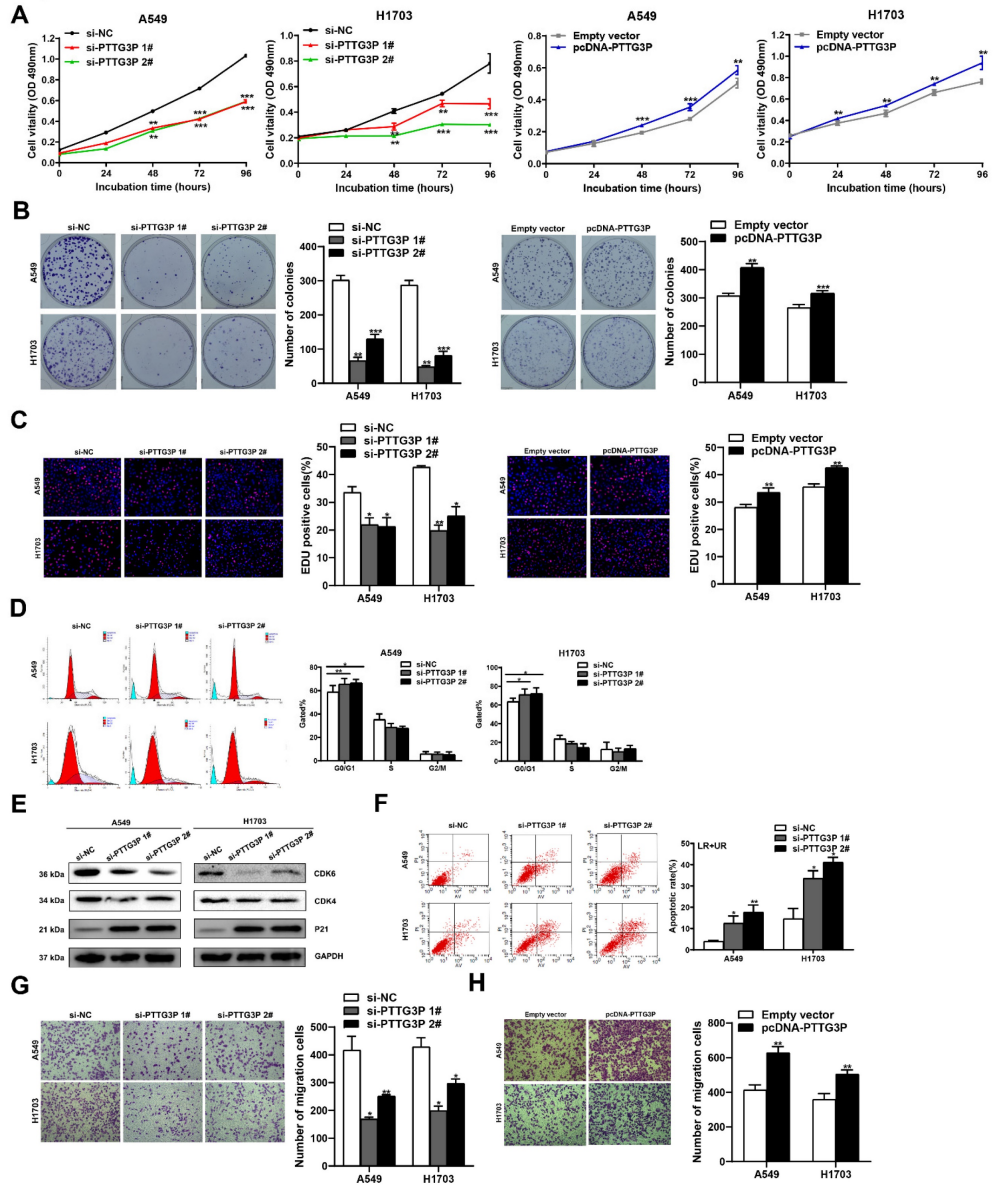
** PTTG3P promotes NSCLC cell proliferation and metastasis* in vitro*. A.** MTT assays were performed to determine the cell viability of transfected A549 and H1703 cells. **B.** Colony formation assays were conducted to determine the proliferation of transfected A549 and H1703 cells. **C.** Proliferating A549 and H1703 cells were labeled with EdU (red). Cell nuclei were stained with Hoechst 33342 (blue). **D.** The cell cycle progression of A549 and H1703 cells transfected with si-PTTG3P were determined by using flow cytometry. **E.** The relative protein expression levels of CDK4/6 and P21 after knockdown of PTTG3P by Western blot. **F.** At 48 h after transfection, flow cytometry was used to investigate the percentage of apoptotic cells of si-PTTG3P-transfected A549 and H1703 cells. LR represents early apoptotic NSCLC cells; UR represents terminal apoptotic NSCLC cells. **G, H.** Transwell assays were used to investigate the alterations to migratory abilities between si-PTTG3P-transfected or pcDNA-PTTG3P-transfected A549 and H1703 cells and control group cells. Representative images and data based on three independent experiments. Error bars indicate mean ± standard errors of the mean. *: p<0.05, **: p<0.01, ***: p<0.001.

To evaluate the biological function of lncRNA PTTG3P in NSCLC cells, we first detected the expression of PTTG3P in a normal human bronchial epithelial cell line (16HBE) and six NSCLC cell lines by qRT-PCR. Compared with 16HBE, lncRNA PTTG3P expression level was significantly upregulated in the six NSCLC cell lines (Fig. S1A). We selected A549 (a LUAD cell line) and H1703 (a LUSC cell line) cell lines for further study. Three individual PTTG3P siRNAs and one specific overexpression plasmid (pcDNA-PTTG3P) were constructed to transfect A549 and H1703 cells, respectively. qRT-PCR detection proved that lncRNA PTTG3P was successfully knocked down or overexpressed in A549 and H1703 cells (Fig. S1B and S1C). We chose si-PTTG3P 1# and si-PTTG3P 2# for the subsequent PTTG3P loss-of-function tests. The effects of lncRNA PTTG3P on the proliferation of NSCLC cells were examined by MTT, EdU, and colony formation experiments. Compared with the control group, cell viability, colony formation efficiency, and the percentage of EdU-positive cells were significantly reduced in A549 and H1703 cells with PTTG3P knockdown, and they were significantly increased in A549 and H1703 cells with PTTG3P overexpression (Fig. 2A-C). To determine the role of PTTG3P in regulating cell cycle progression, propidium iodide (PI) staining, flow cytometry, and Western blots were used to detect the effects of PTTG3P knockdown on cell cycle progression and its regulatory protein levels. The results showed that lncRNA PTTG3P knockdown arrested A549 and H1703 cells in G0/G1 phase, and downregulated the expression levels of cyclin dependent kinase 6/4 (CDK6/4), but significantly upregulated the expression level of p21 (Fig. 2D-E). To clarify the role of lncRNA PTTG3P in the regulation of NSCLC cell apoptosis, flow cytometry was used to detect the effects of PTTG3P knockdown on apoptosis and PI and annexin V double staining were done. The results showed that lncRNA PTTG3P knockdown significantly increased the percentage of apoptosis cells in A549 and H1703 cells (Fig. 2F). These results indicate that lncRNA PTTG3P can act as an oncogene by promoting NSCLC cell proliferation and inhibiting NSCLC cell apoptosis.

### PTTG3P promotes migration and invasion of NSCLC cells

To determine the influence of lncRNA PTTG3P on the migration of NSCLC cells, PTTG3P was knocked down or overexpressed in A549 and H1703 cell lines, and Transwell assays were carried out. The results showed that lncRNA PTTG3P knockdown significantly inhibited the number of migrating cells (Fig. 2G). On the contrary, PTTG3P overexpression significantly promoted cell migration (Fig. 2H). To further investigate the effect of PTTG3P on cell invasion, we performed transwell assays with a matrigel matrix. The results showed that PTTG3P knockdown significantly reduced the number of invaded cells (Fig. S2A), while PTTG3P overexpression significantly promoted cell invasion (Fig. S2B). These results suggest that lncRNA PTTG3P can promote the migration and invasion of NSCLC cells.

### PTTG3P regulates tumorigenesis and metastasis of NSCLC cells *in vivo*

**Figure 3:**
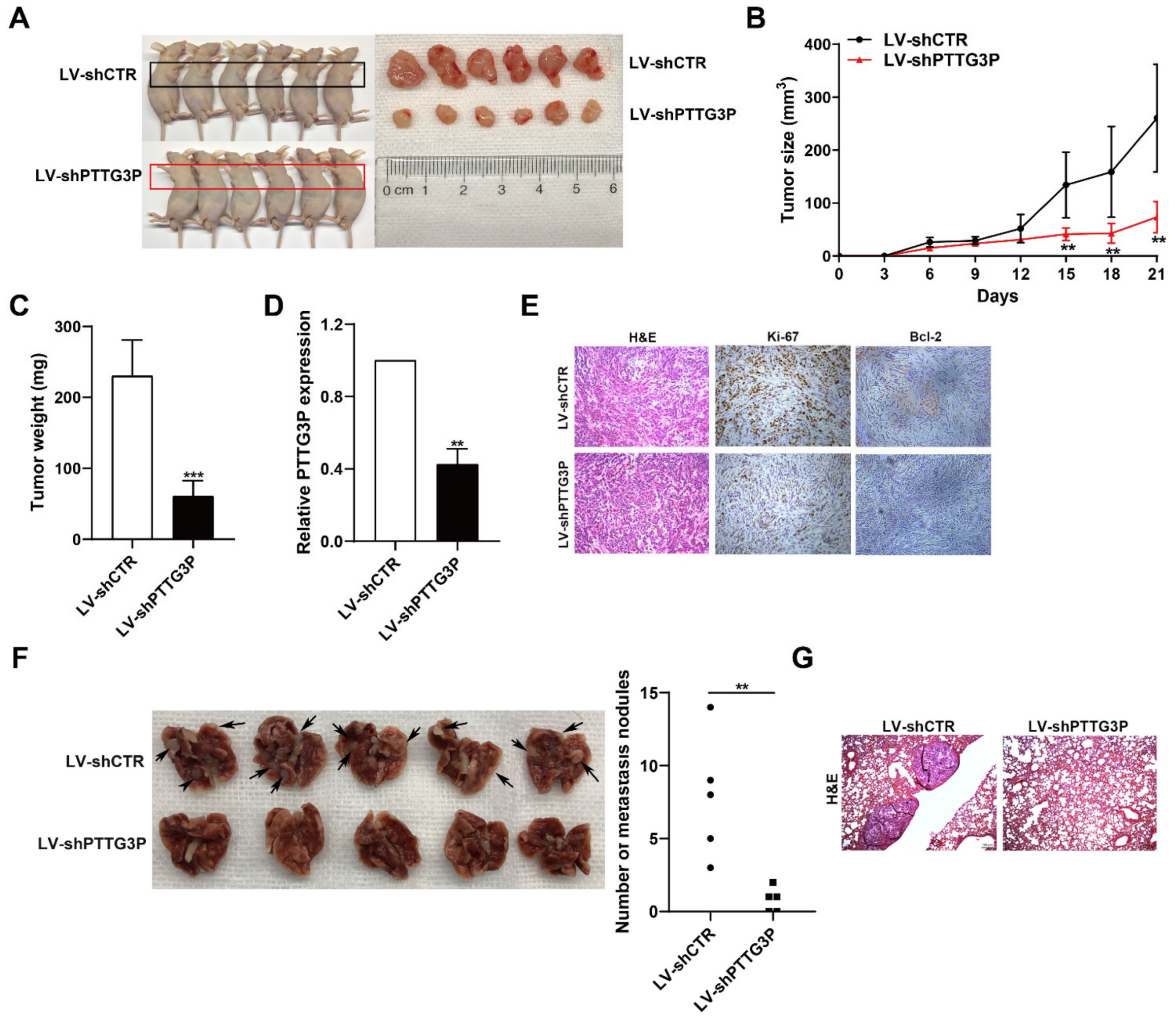
** PTTG3P promotes tumorigenesis and metastasis of A549 cells *in vivo*. A.** LV-shCTR- or LV-shPTTG3P-transfected A549 cells were injected into nude mice (n=6 / group). Tumors that formed in the LV-shPTTG3P group were smaller than the control group. **B.** Tumor volumes were calculated after injection every 3 days. Tumor sizes were measured as length×width^2^ ×0.5. Points, mean (n=6).** C.** Tumor weights were represented as means of tumor weights ± SD.** D.** The expression levels of PTTG3P were detected by qRT-PCR in xenograft tumors (n=6).** E.** The tumor sections were stained by HE staining and IHC staining using antibodies against Ki-67 and Bcl-2.** F.** An experimental metastatic animal model was established by injecting LV-shCTR- or LV-shPTTG3P-transfected A549 cells through the tail vein. Lungs from each experimental group of mice (n=5) showing the numbers of tumor nodules on the lung surface, are shown. **G.** The lung tissue sections were stained using HE stain. Error bars indicate mean ± standard errors of the mean. *: p<0.05, **: p<0.01, ***: p<0.001.

To determine the effect of lncRNA PTTG3P knockdown on tumorigenesis of NSCLC cells *in vivo*, xenograft tumor model was generated by subcutaneously injecting A549 cells stably transfected with LV-shPTTG3P or control vector (LV-shCTR) to male nude mice (n=6); the tumor size was measured once every 3 days to 21 days, and the xenograft tumors were harvested and weighed. The expression of lncRNA PTTG3P in the xenograft tumors was detected by qRT-PCR and the changes to numbers of Ki67- and Bcl-2-positive cells were observed by immunohistochemistry. The results showed that the tumor size and weight at 15-21 days in the PTTG3P knockdown group were significantly lower than those in the control group (Fig. 3A-C). qRT-PCR results showed that the expression level of PTTG3P was significantly lower in the PTTG3P knockdown group than that in the control group (Fig. 3D). H&E staining showed that the xenograft tumors displayed typical characteristics of tumor cells, while the Ki-67- and Bcl-2-positive cells in the PTTG3P knockdown group were significantly lower than those in the control group (Fig. 3E). To determine the effect of lncRNA PTTG3P knockdown on metastasis of NSCLC cells from blood to lung, we injected A549 cells stably transfected with LV-shPTTG3P or LV-shCTR into immunodeficient mice through the tail vein, and established a tumor cell model of blood-lung metastasis. After 8 weeks, the numbers of pulmonary metastatic nodules were counted and H&E staining was performed. The results showed that the numbers of A549 cell metastatic nodules were significantly lower in the PTTG3P knockdown group than that in the control group (Fig. 3F and 3G). These results indicate that lncRNA PTTG3P knockdown not only inhibits the tumorigenesis of NSCLC cells, but also inhibits blood-lung metastasis of NSCLC cells* in vivo*.

To further demonstrate the oncogenic role of PTTG3P in promoting tumorigenesis and metastasis, we have conducted xenograft tumor models and an experimental blood-lung metastasis model using A549 cells overexpressing PTTG3P. The results of these experiments showed that PTTG3P overexpression enhances the tumorigenicity and blood-borne lung metastasis of A549 NSCLC cells *in vivo* (Figs. S3A-G). These *in vivo* data provide stronger support for our conclusions that lncRNA PTTG3P acts as an oncogene by promoting tumor growth and metastasis in NSCLC.

### PTTG3P physically interacts with ILF3

**Figure 4:**
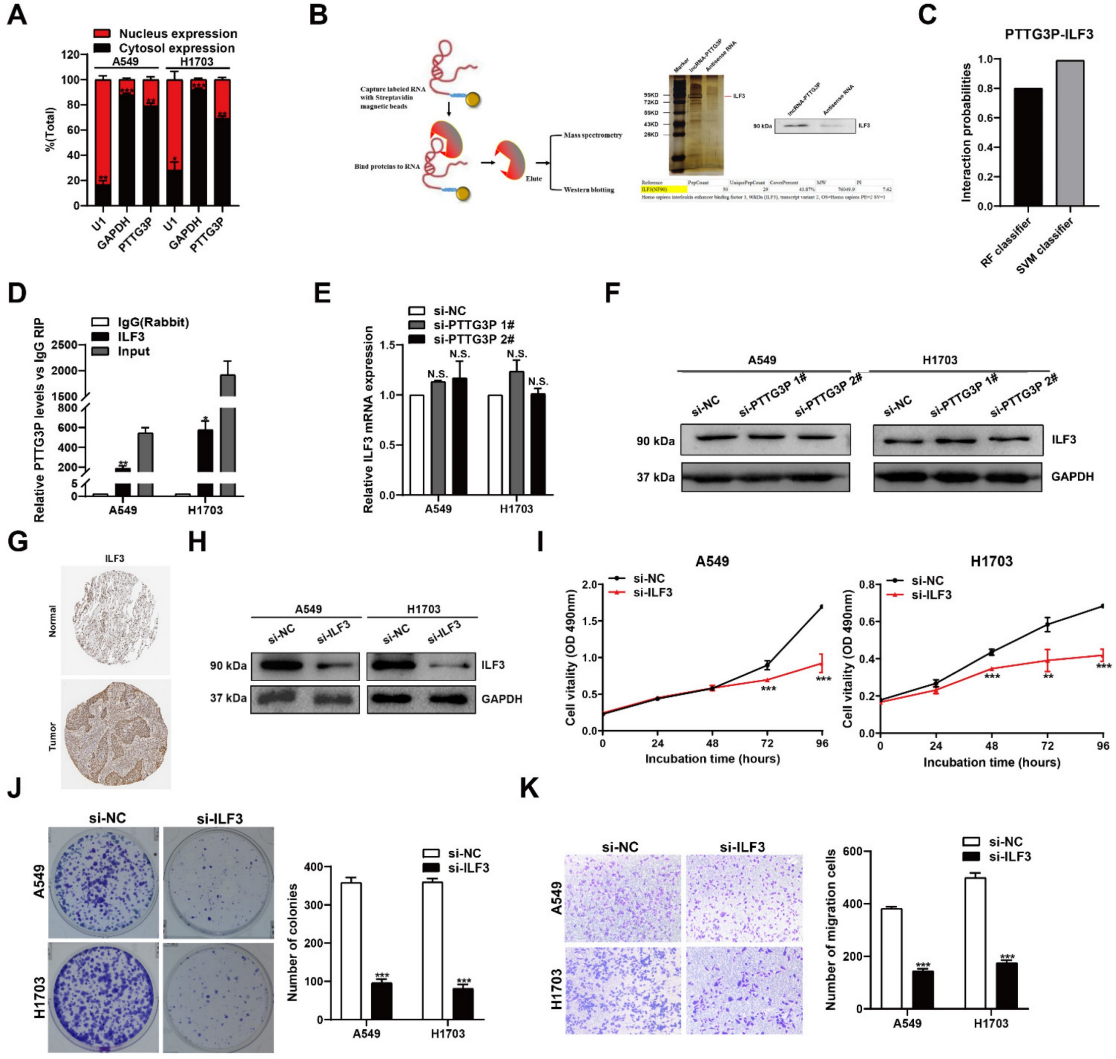
** PTTG3P functions by interacting with ILF3 in the cytoplasm. A.** Nuclear and cytoplasmic RNA isolation analysis of the distribution of PTTG3P in A549 and H1703 cells. RNA expression levels were measured by qRT-PCR. GAPDH was used as a cytosol marker and U1 was used as a nucleus marker.** B.** For the *in vitro* transcribed, pull-down assays-MS and western blot assays showed that desthiobiotinylation-PTTG3P could specifically bind with ILF3 in A549 cells. PTTG3P antisense was used as a control. **C.** The prediction of the interaction probabilities of PTTG3P with RNA-binding protein ILF3 by Bioinformatics (http://pridb.gdcb.iastate.edu/RPISeq/). Predictions with probabilities >0.5 were considered “positive,” indicating that the corresponding RNA and protein are likely to interact with each other. **D.** RIP assays for anti-ILF3 were performed in A549 and H1703 cells and the coprecipitated RNA was subjected to qRT-PCR for lncRNA PTTG3P. The fold enrichment of lncRNA PTTG3P in RIPs is relative to its matching IgG control RIP. **E.** qPCR assay was performed to detect the mRNA expression of ILF3 after PTTG3P knockdown.** F.** Western blot assay showed the ILF3 protein expression levels after PTTG3P knockdown. **G.** Immunohistochemical staining of normal and NSCLC tissues with an anti-ILF3 antibody. **H.** By using Western blot, the protein expression levels of ILF3 were tested in control and si-ILF3-treated-A549 and H1703 cells. **I, J.** MTT and colony formation assays were performed to investigate the changes to the cell viability of si-ILF3-transfected A549 and H1703 cells. **K.** Transwell assays were used to investigate the changes to migratory abilities of NSCLC cells after ILF3 knockdown. Error bars indicate mean ± standard errors of the mean. *: p<0.05, **: P<0.01, ***: p<0.001, n.s.: not significant.

LncRNA PTTG3P was found to be distributed in both the cytoplasm and the nucleus, but was more abundant in the cytoplasm as demonstrated by RNA nuclear and cytoplasmic separation experiments (Fig. 4A), suggesting that it exerts regulatory functions at the post-transcriptional level of genes. The interaction between proteins and lncRNAs, which has been found to be an important mechanism, has been reported to play a key role in cancer development and progress 29. To determine whether lncRNA PTTG3P interacts with proteins to play an important role in NSCLC development and progress, we performed an RNA pull-down assay followed by a proteomic analysis of the PTTG3P-associated protein complex in A549 cells (Fig. 4B, left panel), and protein identity was revealed by mass spectrometry. We found several bands pulled down by vitro-transcribed biotinylated lncRNA PTTG3P sense transcript using silver staining (Fig. 4B, right panel). ILF3, one of the RNA-binding proteins identified by mass spectrometry (MS), was next confirmed by Western blot (Fig. 4B, right panel). The results showed that ILF3 was clearly detected in the PTTG3P pull-down protein complexes but not in antisense PTTG3P. According to Bioinformatics predictions (http://pridb.gdcb.iastate.edu/RPISeq/), the scores for RF Classifier and SVM Classifier were 0.8 and 0.99, respectively (Fig. 4C), supporting the fact that lncRNA PTTG3P binds with ILF3. On the basis of these data, we then performed RNA-binding protein immunoprecipitation (RIP) experiments to investigate PTTG3P and ILF3 binding abundance *in vitro*. We observed lncRNA PTTG3P enrichment using the anti-ILF3 antibody as compared to IgG control in both A549 and H1703 cells. As expected, we found that the ILF3 antibody-bound complex had a significant increase in the amount of PTTG3P compared with the control IgG (Fig. 4D), indicating that PTTG3P may directly interact with the ILF3 protein. However, no changes to ILF3 expression at both mRNA and protein levels in cells with PTTG3P knockdown were observed (Fig. 4E and 4F). Our results proved that lncRNA PTTG3P could bind ILF3 but had no effect on ILF3 expression. Furthermore, we found that the ILF3 protein abundance was increased in the NSCLC specimens from the Human Protein Atlas (www.proteinatlas.org) (Fig. 4G). We next knocked down ILF3 expression in cells (Fig. 4H) and analyzed the effects of ILF3 on NSCLC cell proliferation and cell migration (Fig. 4I-K). The results showed that ILF3 knockdown significantly inhibited the proliferation, colony formation efficiency, and migration of A549 and H1703 cells. Recent studies have reported that ILF3 serves as a post-transcriptional activator, which could form complexes with non-coding RNAs and mRNAs to regulate gene expression and stabilize mRNAs 30, 31. Based on the results presented above, we propose that ILF3 exerts an oncogenic function, and lncRNA PTTG3P promotes cell growth and metastasis, at least in part, in an ILF3-dependent manner.

### PTTG3P promotes MAP2K6 and E2F1 mRNA stability by binding with ILF3

**Figure 5:**
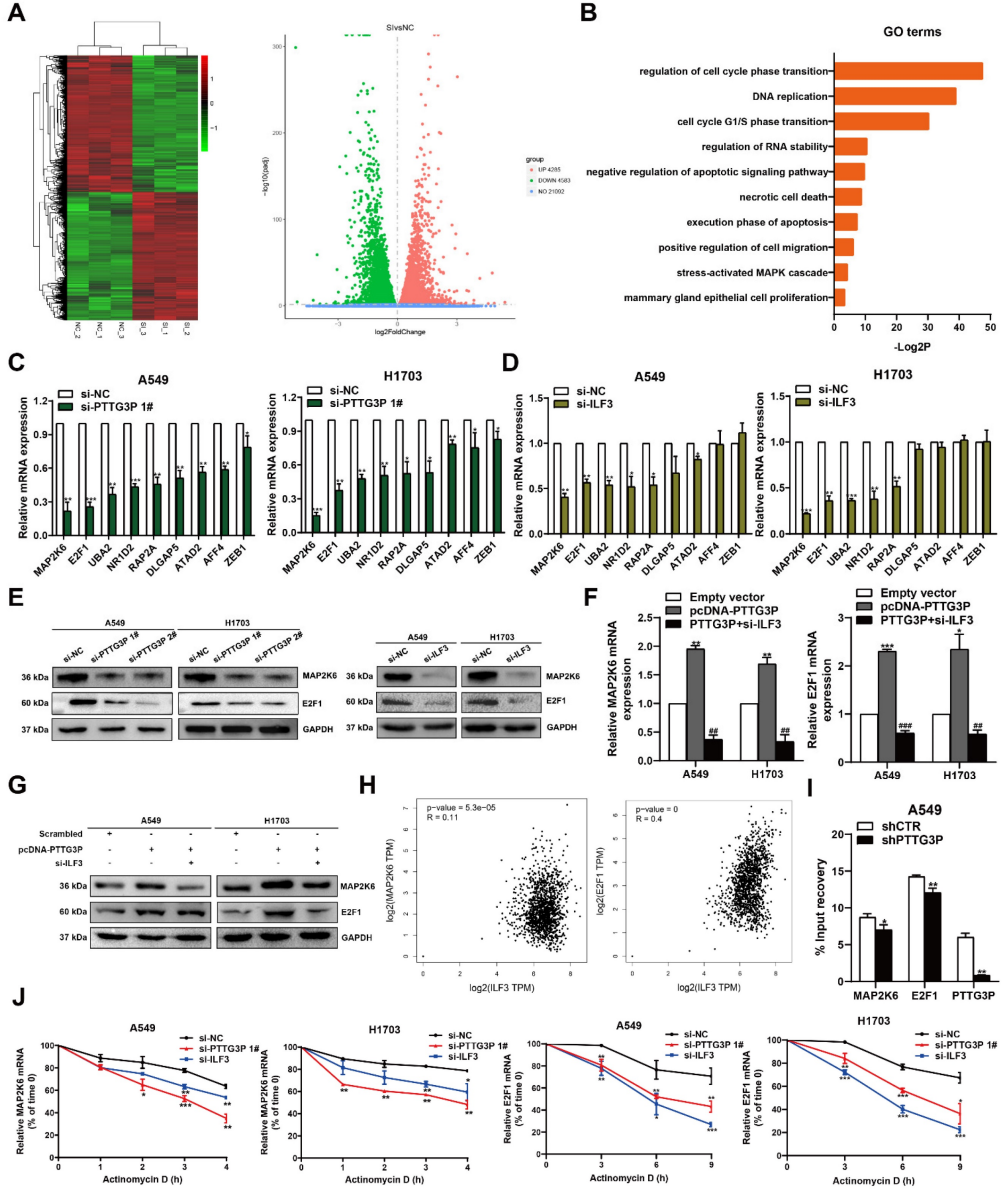
** PTTG3P and ILF3 promote MAP2K6 and E2F1 mRNA stability. A. Left panel,** Mean-centered, hierarchical clustering of gene transcripts altered after knockdown of PTTG3P in A549 cells, with three replicates. **Right panel,** Volcano plots visually show the overall distribution of genes with significant differences in expression. **B.** Gene Ontology (GO) analysis for differentially expressed genes in Fig. 5A. **C, D.** qRT-PCR was performed to validate the altered mRNA levels of candidate genes in PTTG3P- and ILF3-knockdown cells. **E.** Western blot assays detected the protein expression of MAP2K6 and E2F1 following PTTG3P or ILF3 knockdown in A549 and H1703 cells. **F, G.** Based on the qPCR and Western blot assays, the promotion of MAP2K6 and E2F1 (mRNA and protein) by pcDNA-PTTG3P were significantly reversed by the knockdown of ILF3 in A549 and H1703 cells, respectively. **H.** The correlation between the following pairs in NSCLC (N=969) was analyzed using the Gene Expression Profiling Interactive Analysis server: ILF3 and MAP2K6; ILF3 and E2F1. **I.** RIP experiments with anti-ILF3 were performed in shCTR-transfected or shPTTG3P-transfected A549 cells and the coprecipitated RNA was subjected to qRT-PCR for MAP2K6, E2F1, PTTG3P. The fold enrichment in RIPs is relative to its matching IgG control RIP. **J.** For RNA stability determination, Actinomycin D (5 μg/ml) was used to disrupt RNA synthesis in A549 and H1703 cells, and the degradation rates of the MAP2K6 mRNAs were measured every 1 h. The degradation rates of the E2F1 mRNAs were measured every 3 h. Error bars indicate mean ± standard errors of the mean. *: p<0.05, **: p<0.01, ***: p<0.001; ##: p<0.01, ###: p<0.001, compared with pcDNA-PTTG3P-treated cells.

To ascertain the mechanism by which the PTTG3P/ILF3 complex drives the malignant progression of NSCLC and to determine the changes to downstream coding-gene expression levels of PTTG3P, we evaluated the global mRNA effects of PTTG3P knockdown by using RNA transcriptome sequencing (Fig. 5A, left panel). A total of 4,285 genes were significantly upregulated and 4,583 genes downregulated in A549 cells as a consequence of PTTG3P knockdown compared with those in control cells (Fig. 5A, right panel). For RNA-seq results of PTTG3P knockdown, Gene Ontology analysis revealed that the most significantly overrepresented biological processes included pathways involved in cell cycle, apoptosis, and migration (Fig. 5B). The regulation of a subset of target genes was validated by qRT-PCR in PTTG3P knockdown cells independent of those used to generate the RNA-seq data. These representative genes, including key cancer genes that were identified as oncogenes, could play pivotal roles in tumorigenesis. As shown in Fig. 5C, the mRNA levels of MAP2K6, E2F1, UBA2, NR1D2, RAP2A, DLGAP5, ATAD2, AFF4, and ZEB1 were all significantly diminished due to PTTG3P knockdown in both A549 and H1703 cells (Fig. 5C). Then, the role of ILF3 in the coregulation of the suppression of these PTTG3P-suppressed genes was investigated by ILF3 knockdown. Our results showed that MAP2K6 and E2F1 were the most downregulated genes in response to ILF3 downregulation and that siRNA could affect their mRNA expression levels (Fig. 5D). Several studies have demonstrated that aberrant activation of MAP2K6 is involved in specific types of cancers, including human colorectal cancer, esophageal cancer, and cervical cancer 32-34. Among the genes mentioned above, E2F1 was the only transcription factor predicted. E2F1 has been implicated in multiple biological processes and in tumorigenesis, leading potentially to both tumor-promoting as well as tumor-suppressive effects 35. Next, we detected the protein levels of MAP2K6 and E2F1 on knockdown of PTTG3P or ILF3 by using Western blot. Consistent with qRT-PCR, we found that MAP2K6 and E2F1 protein levels were obviously suppressed after PTTG3P or ILF3 knockdown, respectively (Fig. 5E). Furthermore, the increased expressions of MAP2K6 and E2F1 induced by PTTG3P overexpression were reversed by ILF3 knockdown at both the mRNA and protein levels (Fig. 5F and 5G).

We explored the mechanism by which lncRNA PTTG3P regulated MAP2K6 and E2F1 through binding with ILF3, by first doing correlation analysis that showed that E2F1 or MAP2K6 expression levels were positively correlated with ILF3 expression levels in NSCLC tissues from TCGA and/or GTEx data provided by GEPIA (http://GEPIA.cancer-pku.cn/index.html) (Fig. 5H). We next performed RIP assays with anti-ILF3 antibody to predict the binding abundance changes after PTTG3P depletion in A549 cells. The results showed the interactions of ILF3 with the 3´-UTR regions of MAP2K6 and E2F1 mRNAs were decreased following PTTG3P depletion in A549 cells (Fig. 5I). To further investigate whether the PTTG3P-ILF3 complex could regulate the stability of E2F1 or MAP2K6 mRNA, A549 and H1703 cells with PTTG3P or ILF3 knockdown were treated with actinomycin D, respectively, and we determined MAP2K6 and E2F1 mRNA expression levels in the cells every 1 h by using qRT-PCR for a total of four times. The results showed that PTTG3P or ILF3 knockdown significantly reduced the mRNA levels of E2F1 and MAP2K6 at half-life in A549 and H1703 cells compared with the control group (Fig. 5J). These data validated the combination between PTTG3P-ILF3 complex and MAP2K6 or E2F1. We, therefore, concluded that lncRNA PTTG3P could maintain MAP2K6 and E2F1 mRNA stabilities by interacting with ILF3 in NSCLC cells. Based on the ability of E2F1 to transcriptionally upregulate lncRNA, and E2F1, in turn, is co-regulated by PTTG3P and ILF3, we, therefore, suggest that there is an E2F1-PTTG3P-E2F1 positive feedback loop in NSCLC cells, and MAP2K6 may be one of the important downstream target genes of lncRNA PTTG3P.

### The potential reciprocal link between PTTG3P-E2F1 loop

**Figure 6:**
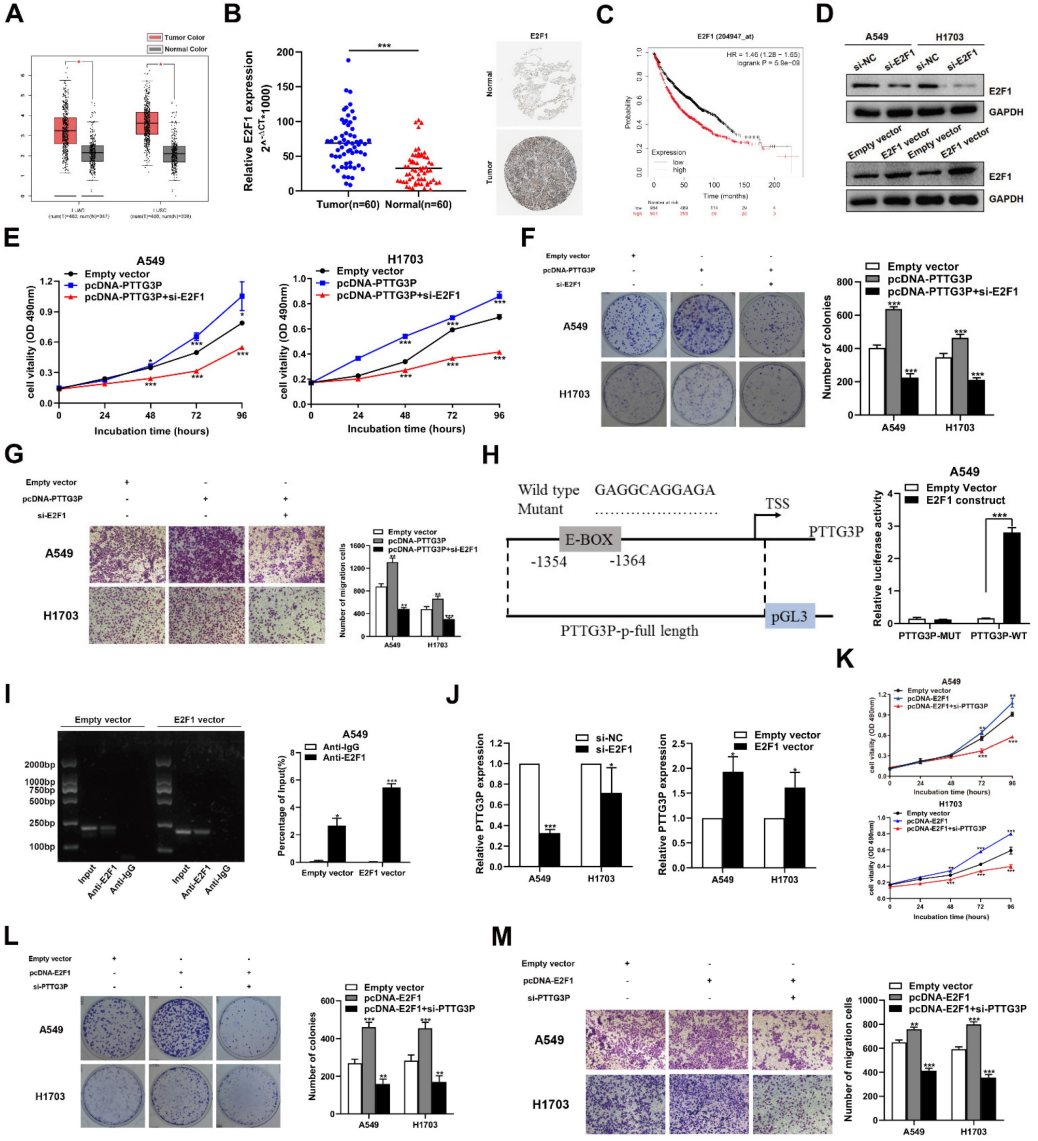
** The potential reciprocal link between PTTG3P-E2F1 loop. A.** E2F1 expression levels in NSCLC from TCGA and/or GTEx data by GEPIA (http://gepia.cancer-pku.cn/index.html) are exhibited, using methods including Pearson, Spearman, and Kendall.** B. Left panel,** E2F1 mRNA expression levels were examined by qRT-PCR in 60 paired human NSCLC tissues and adjacent noncancerous tissues. Data are represented as 2^-△Ct^ *1000 changes. **Right panel,** immunohistochemical staining of normal and NSCLC tissues with an anti-E2F1 antibody.** C.** Kaplan-Meier overall survival curves were analyzed according to online survival analysis software Kmplot (http://www.kmplot.com/lung/). **D.** By using Western blot assays, the relative protein expression levels of E2F1 were tested in control and ectopic E2F1 A549 and H1703 cells.** E-G.** MTT, colony-formation, and Transwell assays were used to determine the cell viability and cell migration abilities of si-E2F1 and pcDNA-PTTG3P co-transfected A549 and H1703 cells. **H. Left panel,** the predicted positions of putative E2F1-binding sites in human PTTG3P promoter by gene sequence analysis (above: wild type sequence) in online data website (JASPAR: http://jaspar.genereg.net/). E2F1-like elements in the PTTG3P promoter region and the E-BOX sequence highlighted as wild type (upper panels). The structure (schematic diagram) of pGL3-PTTG3P promoter reporter plasmid and mutant pGL3-PTTG3P promoter reporter plasmid (lower panels).** Right panel,** the luciferase assays were performed by co-transfecting the PTTG3P promoter fragment (PTTG3P-WT) or deleted PTTG3P promoter fragment (PTTG3P-MUT) with an E2F1 expression construct or an empty vector in A549 cells. Luciferase activity was expressed relative to the activity of pGL3 vector (a promoter-less vector).** I. Left panel,** evaluation of ChIP-qPCR products by agarose gel electrophoresis. **Right panel,** ChIP-qPCR of E2F1 of the promoter region of PTTG3P loci after overexpression of E2F1 in A549 cells. Antibody enrichment was quantified relative to the amount of input DNA. Antibody directed against IgG was used as a negative control.** J.** Analysis of PTTG3P expression levels after transfection of si-E2F1 and overexpression of E2F1 vector in A549 and H1703 cells. **K-M.** MTT, colony formation, and Transwell assays show that the knockdown of PTTG3P could reverse the E2F1-mediated growth and migration promotion. Error bars indicate mean ± standard errors of the mean. *: p<0.05, **: p<0.01, ***: p<0.001.

Among the lncRNA PTTG3P directly-targeted genes mentioned above, E2F1 was the only transcription factor predicted. Moreover, we have confirmed that lncRNA PTTG3P could maintain E2F1 mRNA stability by interacting with ILF3 in NSCLC cells. E2F1 serves as a transcriptional activator and E2F1 activation was associated with cancer progression, including progression in NSCLC 36, 37. We first analyzed the RNA-Seq data of E2F1 expression in NSCLC tissue and adjacent normal tissue samples 38 and the GTEx projects from GEPIA (http://gepia.cancer-pku.cn/). E2F1 expression was clearly upregulated in the paired and unpaired NSCLC tissues (*p*<0.05) (Fig. 6A). We hypothesized the potential role of E2F1 as an oncogene in NSCLC tissues. In support of this possibility, we found that the mRNA and protein expression levels of E2F1 in the tumor tissues were higher than those in the paracancerous tissues (Fig. 6B). Importantly, the Kaplan-Meier analysis of patient survival showed that E2F1 expression was negatively correlated with overall survival (Fig. 6C). To further evaluate the roles of E2F1 in PTTG3P expression, we used an overexpression plasmid of E2F1 and siRNA targeting of E2F1 to upregulate or downregulate the expression of E2F1 in A549 and H1703 cells (Fig. 6D). By using MTT and colony formation assays, we found that knockdown of E2F1 induced diminished effects in proliferation promoted by PTTG3P overexpression in A549 and H1703 cells (Fig. 6E and 6F). We also found that the knockdown of E2F1 reversed the PTTG3P-induced cell migration (Fig. 6G).

Considering that E2F1 serves as a transcriptional activator, interestingly, we found a conserved E2F1 binding site at the promoter region of lncRNA PTTG3P (2 kb upstream of the transcription start site [TSS]) by using an online website (JASPAR: http://jaspar.genereg.net/) combined with the UCSC Genome Bioinformatics Site (http://genome.ucsc.edu/) (Fig. 6H, left panel). Similar to mRNA transcription, the transcription process of lncRNA is also regulated by epigenetic processes and many transcription factors 39, 40. To explore the potential interaction between PTTG3P and E2F1 in NSCLC, we deleted the E-box binding site and carried out the luciferase reporter assay. The results showed that the deletion of the E2F1-binding motif (an enhancer box, E-BOX) significantly impaired the effect of E2F1 on PTTG3P transcription activation, suggesting that E2F1 binds to special binding motifs to regulate lncRNA PTTG3P transcription in A549 cells. The PTTG3P promoter-driven firefly expression vector enhanced PTTG3P transcriptional activity via E2F1 overexpression (E2F1 construct) (Fig. 6H, right panel). Furthermore, the ChIP assays confirmed that E2F1 directly binds to the promoter region of PTTG3P, and that the overexpression of E2F1 enhanced the strength of this binding (Fig. 6I). Subsequently, we knocked down E2F1 by qRT-PCR analysis in NSCLC cells, which resulted in a significant downregulation of PTTG3P expression. Conversely, after overexpression of E2F1, PTTG3P expression levels were significantly upregulated (Fig. 6J). Therefore, we proposed a hypothesis that E2F1-PTTG3P forms a positive feedback loop. To investigate the interaction of lncRNA PTTG3P in E2F1-overexpression NSCLC, PTTG3P expression was knocked down by siRNA in E2F1-overexpression A549 and H1703 cells. The results showed that the enhanced cell viability, clonogenicity, and migration capability induced by E2F1 overexpression were markedly inhibited by knockdown of lncRNA PTTG3P (Fig. 6K-M). Based on these data, we confirmed that E2F1 binds to the PTTG3P promoter region and subsequently enhances its transcriptional activity, indicating that PTTG3P/E2F1 formed a positive feedback loop.

### MAP2K6 is involved in the PTTG3P-induced NSCLC cell proliferation and migration

**Figure 7:**
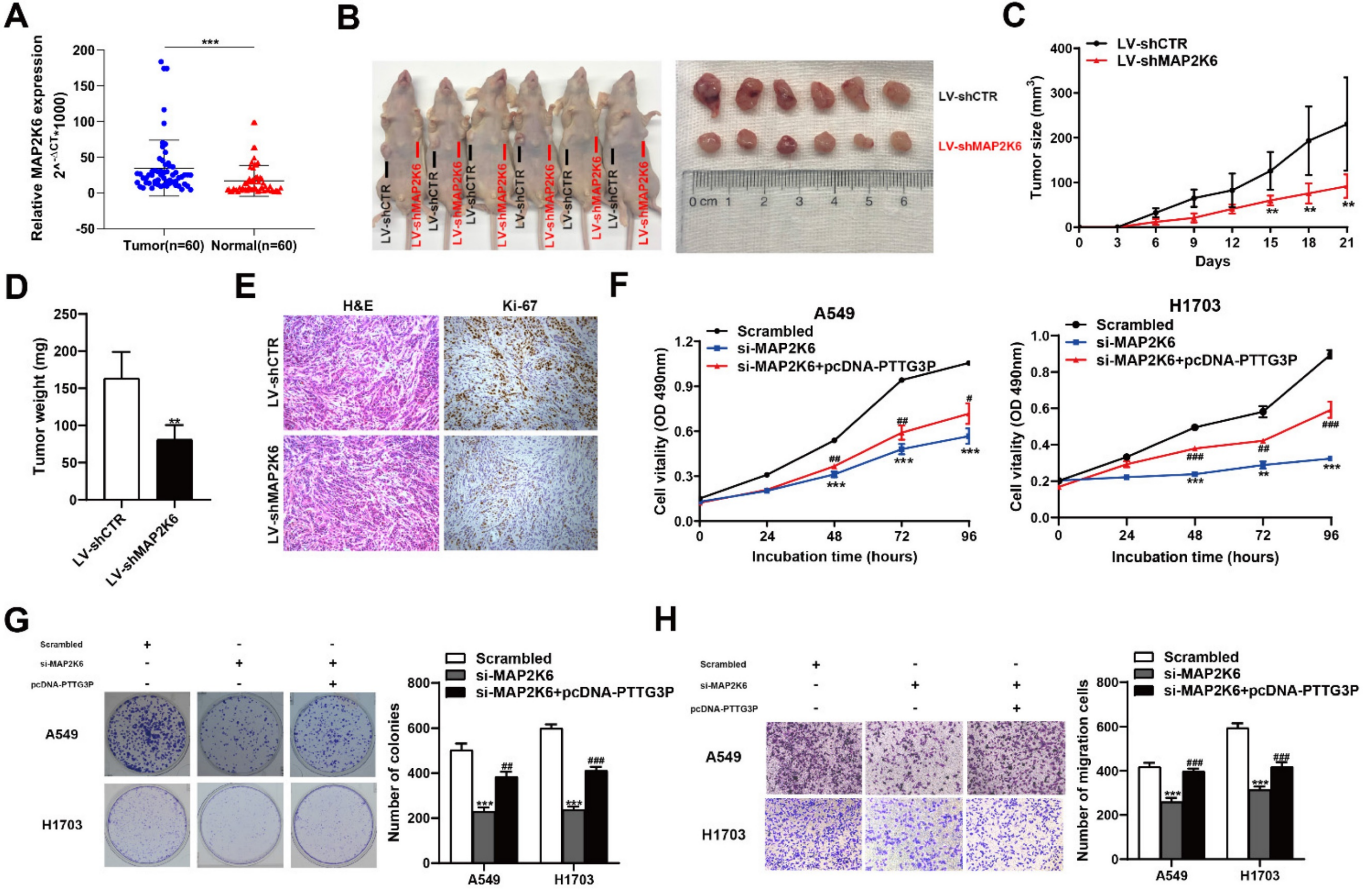
** MAP2K6 is involved in the PTTG3P-induced NSCLC cell proliferation and migration. A.** Relative expression of MAP2K6 in 60 paired human NSCLC tissues and the corresponding adjacent non-tumor tissues were analyzed by qRT-PCR and normalized against GAPDH expression. Data are represented as 2^-△Ct^ *1000 changes.** B.** LV-shCTR or LV-shMAP2K6 stably transfected into A549 cells, which were injected into nude mice (n=6). **C.** Tumor sizes were measured every three days after the subcutaneous tumor formed. **D.** Up to 21 days after injection, tumor weights were recorded, and the average tumor weight in the LV-shMAP2K6 group was significantly lower than the LV-shCTR group.** E.** Representative images of HE staining and IHC staining using antibodies against Ki-67 of the subcutaneous tumor.** F, G.** MTT and colony formation assays were used to determine the cell viability of si-MAP2K6- and pcDNA-PTTG3P-co-transfected A549 and H1703 cells. **H.** Transwell assays show that PTTG3P overexpression could reverse si-MAP2K6-mediated cell metastasis inhibition. Error bars indicate mean ± standard errors of the mean. *: p<0.05, **: p<0.01, ***: p<0.001; #: p<0.05, ##: p<0.01, ###: p<0.001, compared with pcDNA-PTTG3P-treated cells.

MAP2K6, also known as MEK6 or MKK6, functions as a mitogen-activated protein (MAP) kinase and acts as an integration point for multiple biochemical signals. As an essential component of the p38 MAP kinase-mediated signal transduction pathway, this gene is involved in many cellular processes, such as stress-induced cell cycle arrest, transcription activation, and apoptosis 41-43. We first examined the tumor/normal differential expression of MAP2K6 by qRT-PCR assays in 60 paired NSCLC samples and paracancerous tissues. The results showed that MAP2K6 expression levels were significantly upregulated in 60 pairs of NSCLC tissues compared with those in paracancerous tissues (Fig. 7A). To confirm the role of MAP2K6 in tumor growth, LV-shCTR or LV-shMAP2K6 were stably transfected into A549 cells, which were injected into nude mice, respectively (n=6) (Fig. 7B). Tumor volumes and tumor weights were measured. *In vivo* experiments revealed that tumor growth in the LV-shMAP2K6 group was obviously slower than that in the LV-shCTR group (Fig. 7C and 7D). Immunohistochemistry analysis further confirmed that the tumors that developed from LV-shMAP2K6 transfected-cells displayed lower Ki-67 staining than the control group (Fig. 7E). Importantly, we also found that knockdown of MAP2K6 induced the suppression of proliferation and migration in A549 and H1703 cells in MTT (Fig. 7F), colony formation (Fig. 7G), and Transwell assays (Fig. 7H). In addition, PTTG3P overexpression could reverse si-MAP2K6-mediated growth and migration suppression (Fig. 7F-H). Our results confirmed that MAP2K6 is involved in the lncRNA PTTG3P-induced NSCLC cell proliferation and migration.

**Figure 8:**
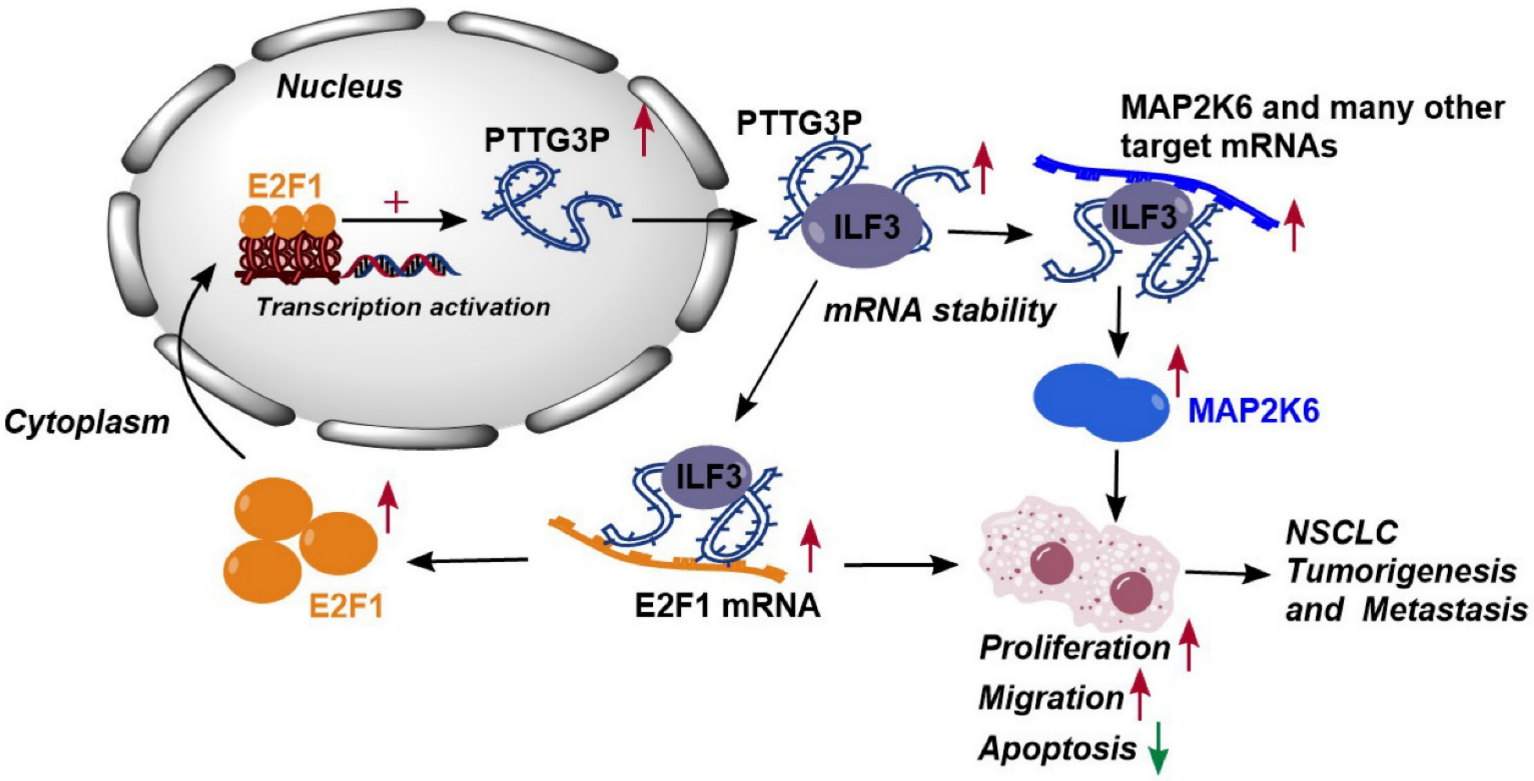
** Proposed model in which PTTG3P mediates the proliferation and metastasis progression of NSCLC.** Abnormal E2F1-mediated activation of lncRNA PTTG3P could promote NSCLC cell proliferation and migration partially by directly forming an RNA-protein complex with ILF3 and then stabilizing MAP2K6 and E2F1 mRNA stabilities in NSCLC cells. PTTG3P was involved in a positive feedback loop with E2F1 and forming the E2F1/PTTG3P/ILF3/MAP2K6 cell axis.

Altogether, these results demonstrated that abnormal E2F1-mediated activation of lncRNA PTTG3P exhibited potent effects on NSCLC progression. lncRNA PTTG3P was essential for promoting NSCLC cell growth and metastasis by binding to ILF3, which stabilizes the expression of MAP2K6 and E2F1. Collectively, this study provides mechanistic insights into the roles of lncRNA PTTG3P in NSCLC, such as by forming the E2F1/PTTG3P/ILF3/MAP2K6 signaling axis and a positive feedback loop between PTTG3P and E2F1 (Fig. 8). We hereby emphasize that lncRNA PTTG3P might be used as a biomarker and therapeutic target for diagnosis and treatment of NSCLC.

## Discussion

Although there are a few well-characterized lncRNAs in NSCLC, many lncRNAs remain un-characterized and their mechanisms of action are largely unknown 9. To investigate the potential biological functions and underlying mechanistic details for lncRNAs in NSCLC, we identified a lncRNA highly expressed in NSCLC from GSE74706 datasets, lncRNA PTTG3P. Then, we harvested 60 paired NSCLC tissues and paracancerous tissues and verified that the expression level of lncRNA PTTG3P was markedly upregulated in NSCLC tissues using qRT-PCR. Furthermore, we found that the high expression of lncRNA PTTG3P in NSCLC positively correlated with an advanced TNM stage, a larger tumor size, and poor survival of NSCLC patients. Similar results were also reported recently 23. In that study, the authors analyzed five public datasets (GSE18842, GSE19804, GSE27262, GSE30219, and GSE19188), and found that lncRNA PTTG3P was significantly upregulated in NSCLC samples, and positively correlated with pathological stage and poor survival of NSCLC patients. These results suggest that lncRNA PTTG3P might play an essential role in NSCLC progression. However, the biological function and potential molecular mechanism of action of lncRNA PTTG3P in NSCLC have not been investigated in the previous study.

To investigate the relationship between the lncRNA PTTG3P and its protein-coding gene family members PTTG1 and PTTG2 in NSCLC, we examined how modulating PTTG3P levels affects the expression of PTTG1 and PTTG2. We measured PTTG1 and PTTG2 mRNA levels after knocking down or overexpressing PTTG3P in NSCLC cells. Knocking down PTTG3P significantly decreased PTTG1 expression but did not affect PTTG2 levels. In contrast, PTTG3P overexpression increased PTTG1 expression while PTTG2 remained unchanged (Results not shown). These results indicate PTTG3P specifically regulates the expression of the oncogenic PTTG1 pseudogene family member in NSCLC cells, but does not affect PTTG2. The homologous sequence between PTTG3P and PTTG1 suggests direct competing endogenous RNA regulatory mechanisms may be involved.

Recently lncRNA PTTG3P has been reported to stimulate cancer cell proliferation and migration in gastric cancer 15, hepatocellular carcinoma 16, cervical cancer 17, breast cancer 18, colorectal cancer 19, and pancreatic cancer 20 and to inhibit cancer cell apoptosis in gastric cancer 15 and hepatocellular carcinoma 16. Bioinformatics analysis showed that lncRNA PTTG3P was associated with NSCLC cell proliferation 23. To explore the biofunction of lncRNA PTTG3P in NSCLC, we performed loss-of- and gain-of-function experiments in NSCLC cell lines. Our results demonstrated that lncRNA PTTG3P expression level was significantly upregulated in 6 NSCLC cell lines. Knockdown of lncRNA PTTG3P markedly inhibited cell proliferation and cell cycle progression with downregulation of CDK4/6 and upregulation of p21, and migration, but significantly increased percentage of cell apoptosis. In contrast, overexpression of lncRNA PTTG3P significantly increased cell proliferation and migration. Xenograft mouse models demonstrated that lncRNA PTTG3P knockdown not only inhibits the tumorigenesis of NSCLC cells, but also inhibits the blood-lung metastasis of NSCLC cells* in vivo*. We also have conducted xenograft tumor models and an experimental blood-lung metastasis model using A549 cells overexpressing PTTG3P. Our results indicate that PTTG3P overexpression enhances the tumorigenicity and blood-borne lung metastasis of A549 NSCLC cells *in vivo*. These *in vivo* data provide stronger support for our conclusions that lncRNA PTTG3P acts as an oncogene by promoting tumor growth and metastasis in NSCLC.

Given that lncRNA PTTG3P is a predominantly cytoplasm-located lncRNA, most mechanistic studies on lncRNA PTTG3P in cancer initiation and progression were focused on it serving as competing endogenous RNA through competitively binding to shared miRNAs 16, 18-20, 26. Bioinformatics analysis identified 10 miRNAs (hsa-miR-129-5p, hsamiR-3167, hsa-miR-376c-3p, hsa-miR-132-3p, hsamiR-212-3p, hsa-miR-383-5p, hsa-miR-876-5p, hsa-miR-873-5p, hsa-miR-421, hsa-miR-505-3p) as key regulators in PTTG3P-related ceRNA networks in NSCLC 23. Besides the ceRNA mechanism, the interaction between proteins and lncRNAs, has been found to be a key mechanism, and has been reported to play an important role in cancer development and progress 29. To determine whether PTTG3P interacts with proteins to play an important role in NSCLC development and progression, we used RNA pull-down assay and protein mass spectrometry analysis and demonstrated that PTTG3P could bind to ILF3 protein. Bioinformatics (http://pridb.gdcb.iastate.edu/RPISeq/) predicted that PTTG3P could bind to ILF3, and RNA-binding protein immunoprecipitation (RIP) experiments confirmed the mutual binding of PTTG3P and ILF3. However, PTTG3P knockdown did not alter the expression levels of ILF3 mRNA and protein in NSCLC cells. Our results demonstrated that PTTG3P could bind ILF3, but had no effect on ILF3 expression.

It has been shown that various combinations of interactions between lncRNA and proteins regulate cancer phenotypes via modulation of different target proteins. However, the interactions between the lncRNAs and the target proteins have not been well-studied as yet 29. A previous study has revealed that ILF3 is involved in the mediators of mRNA translational regulation, stability, and subcellular localization by acting as an RNA binding protein 44. Several works support the role of lncRNA-ILF3 interactions as an enhancer of mRNA stability 38. To determine the role of ILF3 in mediating PTTG3P in NSCLC progression, we examined the expression of ILF3 in NSCLC tissues and the effect of ILF3 knockdown on NSCLC cell proliferation and migration. The results revealed that the expression level of ILF3 was significantly upregulated in NSCLC tissues, and knockdown of ILF3 significantly inhibited the proliferation and migration of NSCLC cells. To identify the common downstream targets of the PTTG3P/ILF3 complex and the mechanisms driving the malignant progression of NSCLC, we used RNA transcriptome sequencing, bioinformatics analysis, and qRT-PCR validation to find that MAP2K6 and E2F1 expression levels were evidently downregulated in PTTG3P-knockdown NSCLC cells; simultaneously, qRT-PCR assay also found that the expression levels of MAP2K6 and E2F1 mRNAs were significantly downregulated in ILF3-knockdown NSCLC cells. The protein expression levels of MAP2K6 and E2F1 were significantly downregulated in PTTG3P or ILF3 knockdown NSCLC cells, and ILF3 knockdown could also significantly downregulate the mRNA and protein expression levels of MAP2K6 and E2F1 caused by overexpression of PTTG3P in NSCLC cells. Bioinformatics analysis revealed that E2F1 or MAP2K6 expression levels were positively correlated with ILF3 expression levels in NSCLC tissues. The results of RIP assay using anti-ILF3 antibody showed that the interaction of ILF3 with the 3'-UTR regions of MAP2K6 and E2F1 mRNA was reduced in NSCLC cells with PTTG3P depletion. Furthermore, we demonstrated that knockdown of PTTG3P or ILF3 in NSCLC cells significantly reduced the mRNA stability of E2F1 and MAP2K6. The results of this study demonstrated that PTTG3P maintains the stability of MAP2K6 and E2F1 mRNA at the post-transcriptional level by binding to ILF3.

The transcription factor E2F1 plays a crucial role in mediating multiple cancer hallmark capabilities that regulate cell cycle, survival, apoptosis, metabolism, and metastasis 45. Dysregulation of E2F1 has been demonstrated in a number of cancers, including lung cancer 46-51. In the present study, we analyzed the expression level changes to E2F1 in NSCLC tissues and adjacent tissues from the GTEx project (http://gepia.cancer-pku.cn/) 48 and examined the expression level changes to E2F1 in 60 pairs of NSCLC tissues and paracancerous tissues harvested by us by qRT-PCR assay. The results showed that the expression level of E2F1 was significantly upregulated in NSCLC tissues, and the expression of E2F1 was negatively correlated with the overall survival rate. Furthermore, we knocked down E2F1 in PTTG3P-overexpressing NSCLC cells, and the results showed that E2F1 knockdown could significantly inhibit the PTTG3P overexpression-induced NSCLC cell growth acceleration and migration increase. Our results suggest that E2F1 not only plays an important role in NSCLC development, but also plays a key role in mediating PTTG3P-induced NSCLC development.

In recent studies, it has been demonstrated that E2F1 can function as a transcription factor to regulate the expression of lncRNA in gastric cancer 52, cervical cancer 53, pituitary adenomas 54, and NSCLC 47, 50. However, it is unclear whether E2F1 could regulate PTTG3P expression at the transcriptional level. To answer this question, we completed bioinformatics analysis and found a binding site between E2F1 and the promoter region of PTTG3P. Luciferase assay and ChIP assay demonstrated that E2F1 could regulate PTTG3P expression at the transcriptional level. We also found that E2F1 knockdown significantly downregulated the expression level of PTTG3P, while E2F1 overexpression significantly upregulated the expression level of PTTG3P in NSCLC cells and stimulated the proliferation and migration of NSCLC cells, which were inhibited by PTTG3P knockdown. Altogether, our results indicate that a positive feedback loop composed of PTTG3P, ILF3, and E2F1 was responsible for PTTG3P-mediated NSCLC development and metastasis.

MAP2K6, also known as MKK6, is a key factor in MAP2K6-p38 signaling and is related to cancer cell proliferation, cell cycle progression, and tumorigenicity 41. MAP2K6 plays a dual role by either promoting or suppressing cancer depending on cell-type and context 55. MAP2K6 is overexpressed in esophageal, stomach, and colon cancer 41 and increased expression has been observed in prostate cancer upon progression 56. However, it is not known whether MAP2K6 plays a role in NSCLC development by either promoting or suppressing cancer. To answer this question, we examined the expression level changes to MAP2K6 in 60 pairs NSCLC tissues and paracancerous tissues by qRT-PCR assay and observed the effects of MAP2K6 knockdown on NSCLC growth by xenograft experiments. Our results showed that the expression level of MAP2K6 was significantly upregulated in NSCLC tissues, and MAP2K6 knockdown inhibited the growth of xenografts by significantly inhibiting the proliferation of NSCLC cells. Furthermore, we overexpressed PTTG3P in MAP2K6 knockdown NSCLC cells. The results showed that PTTG3P overexpression could partially correct the inhibition of NSCLC cell growth and migration caused by MAP2K6 knockdown. Our findings indicate that MAP2K6 not only plays an important role in NSCLC development, but also plays a significant role in mediating PTTG3P-induced NSCLC development.

In conclusion, the results of this study indicate that lncRNA PTTG3P upregulates the protein expression levels of MAP2K6 and E2F1 by binding to ILF3 to maintain the mRNA stability of MAP2K6 and E2F1, while E2F1 transcriptionally upregulates lncRNA PTTG3P to form a PTTG3P/ILF3/E2F1 positive feedback loop, which in turn promotes NSCLC occurrence and metastasis (Fig. 8). This study not only reveals a novel mechanism of action of lncRNA PTTG3P in promoting the occurrence and metastasis of NSCLC, but also provides an experimental basis for the use of PTTG3P, ILF3, E2F1, and MAP2K6 as biomarkers and therapeutic targets for the diagnosis and treatment of NSCLC.

## Supplementary Material

Supplementary figures and tables.
