# Supplementary material for: LncRNA PTTG3P promotes tumorigenesis and metastasis of NSCLC by binding with ILF3 to maintain mRNA stability and form a positive feedback loop with E2F1
Source: Int J Biol Sci. 2023 Aug 21;19(13):4291–310. doi: 10.7150/ijbs.81738 (PMC10496499; doi:10.7150/ijbs.81738)
Supplement: Supplementary file 1 — Supplementary figures and tables. [file ijbsv19p4291s1.pdf]

Supplementary information

Figure S1

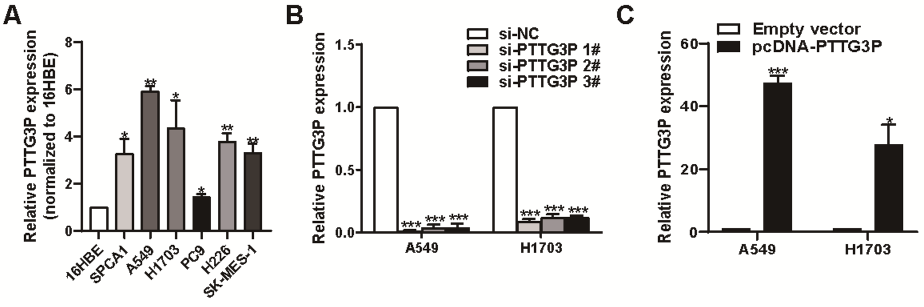

Figure S1. The expression levels of PTTG3P in NSCLC cells. A. qRT-PCR analysis of PTTG3P expression in the normal lung bronchial epithelial cell line (16HBE) and six NSCLC cells. **B.** Relative expression levels of PTTG3P in A549 and H1703 cells transfected with si-NC or si-PTTG3P 1#, 2#, 3#. **C.** qRT-PCR analysis of PTTG3P expression levels following the treatment with empty vector or pcDNA-PTTG3P. *:P<0.05, **:P<0.01, ***:P<0.001.

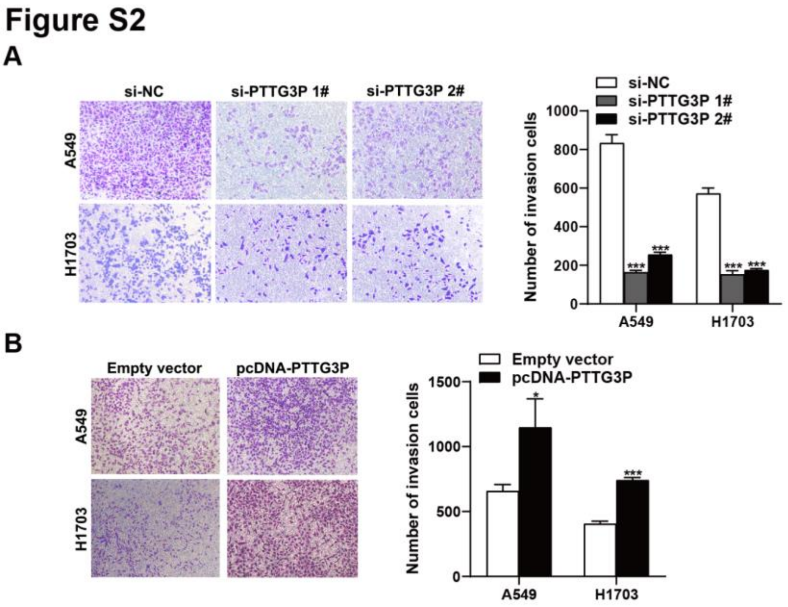

Figure S2. Cell invasion assay was used to determine the cell invasive capacity with Matrigel after PTTG3P knockdown or overexpression. A, B. The number of invasion cells were determined by using Transwell assays with Matrigel matrix (30ug) in PTTG3P-treated A549 and H1703 cells. All experiments were conducted in biologic triplicates with three technical replicates. Data are presented as mean ± SD. *P<0.05, ***P<0.001.

Figure S3

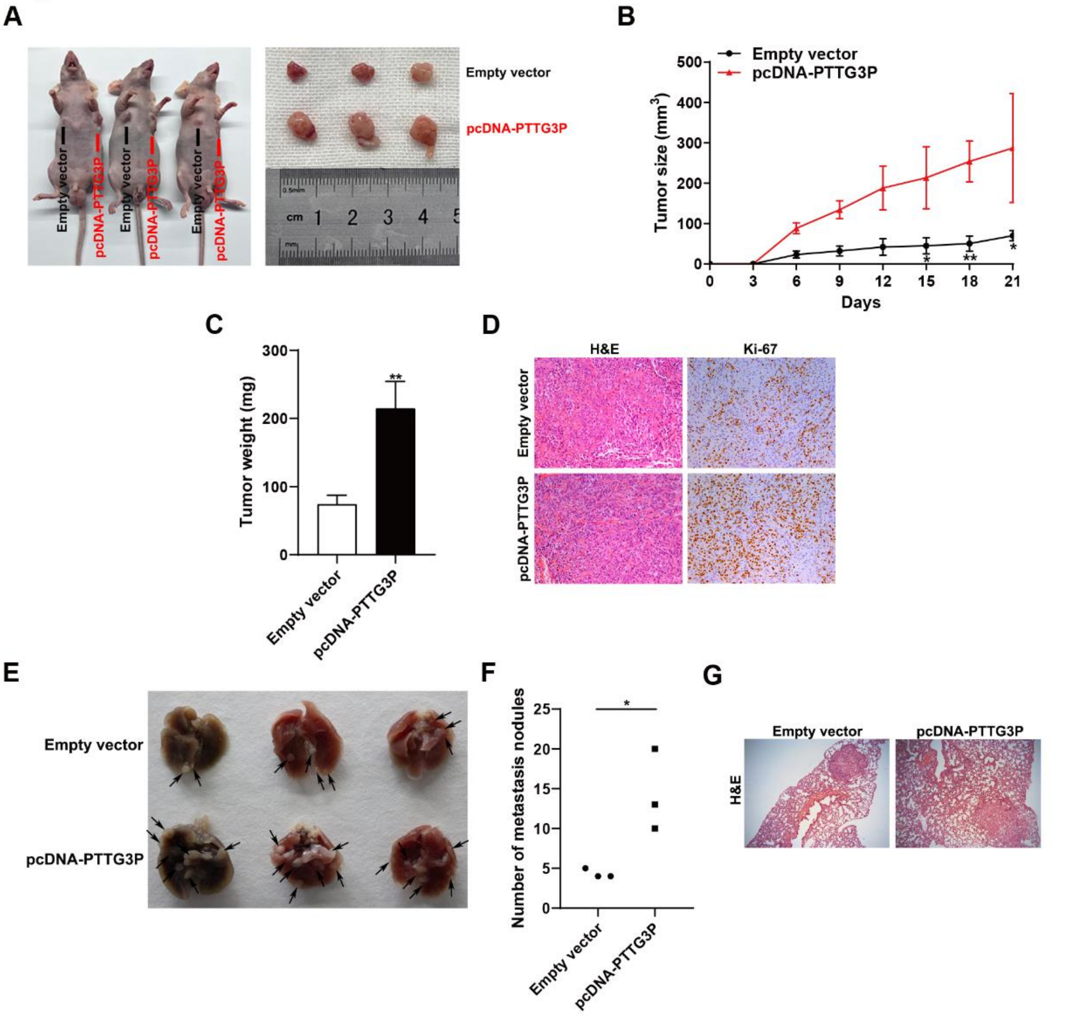

Figure S3. PTTG3P promotes tumorigenesis and metastasis of A549 cells *in vivo*. **A.** Empty vector or pcDNA-PTTG3P transfected A549 cells were injected subcutaneously into the nude mice (n = 3), respectively. **B.** Tumor volumes were calculated after injection every three days. Tumor sizes were measured with length×width^2 ×0.5. (n = 3). **C.** Tumor weights were represented as means of tumor weights ± SD. **D.** The tumor sections were under H&E staining and IHC staining using antibodies against Ki-67. **E, F.** An experimental metastatic animal model was established by injecting empty vector or pcDNA-PTTG3P transfected A549 cells through the tail vein. Lungs from each experimental group of mice (n = 3) showing the

numbers of tumor nodules on the lung surface, were shown. **G.** The lung tissue sections were under H&E staining. Error bars indicate mean ± standard errors of the mean. *P<0.05, **P<0.01.

Supplemental Table S1. The clinic-pathological characteristics of NSCLC patients.

**Characteristics**	**(%) of patients**	**Expression of PTTG3P**		**P value [a]**
		**Low (n=30)**	**High(n=30)**	
**Sex**				1
Male	48 (80%)	24	24	
Female	12 (20%)	6	6	
**Age**				0.017*
≤60	23 (38.3%)	7	16	
>60	37 (61.7%)	23	14	
**Tumor grade**				0.083
Middle to Low	50 (83.3%)	22	28	
High	10 (16.7%)	8	2	
**TNM stage**				
Ⅰ and Ⅱ	35 (58.3%)	27	8	0.001*
Ⅲ and Ⅳ	25 (41.7%)	3	22	
**Tumor size**				0.017*
≤3 cm	15 (25%)	12	3	
>3 cm	45 (75%)	18	27	
**Lymph node metastasis (N)**				0.001*
N0	21 (35%)	19	2	
N1 or above	39 (65%)	11	28	
**History of smoking**				0.573
Ever	18 (30%)	10	8	
Never	42 (70%)	20	22	

[a]: For analysis of correlation between lncRNA PTTG3P levels and clinical featureas, Pearson's Chi-square tests were used. Fisher's exact tests was used when the expected count of variable was less than 5. *:P < 0.05.

Supplemental Table S2. Sequences of primers used in this study.

Gene	Forward primer (5'-3')	Reverse primer (5'-3')
GAPDH	GGGAGCCAAAAGGGTCAT	GAGTCCTTCCACGATACCAA
PTTG3P	TCTGGTTGAGAGCGGCAATA	TCAGCCCATCCTTTGTAGCC
U1	GGGAGATACCATGATCACGAAGGT	CCACAAATTATGCAGTCGAGTTTCCC
ILF3	AACCATGGAGGCTACATGAAT	CGCTCTAGGAAGACCCAAAATC
E2F1	AGCGGCGCATCTATGACATC	GTCAACCCCTCAAGCCGTC
MAP2K6	TCAATGCTCTCGGTCAAGTG	ATGCCCAGACTCCAAATGTC
PTTG3P Promoter primer 1	GAGAATCACTTGAACCTG	TATAGATGTTGCTCCACTT
PTTG3P Promoter primer 2	AAACCCTGACTCTACTAAA	GTTCAAGTGATTCTCCTG
3'RACE-F1	TGCCCCACCAGCCTTACCTAAAGC	
3'RACE-F2	TGCCAAAAAGATGACTGAGAAGAC	
5'RACE-R1		ATGGTGGAGAGGGCATCTTCACAG
5'RACE-R2		AGGCATCATCTGAGGCAGGAACAG

Abbreviation:

GAPDH, glyceraldehyde-3-phosphate dehydrogenase;

PTTG3P, pituitary tumor-transforming 3, pseudogene;

U1, U1 small nuclear RNA;

ILF3, interleukin enhancer binding factor 3;

E2F1, E2F transcription factor 1;

MAP2K6, mitogen-activated protein kinase kinase 6.

RACE, rapid amplification of cDNA end.

Supplemental Table S3. Sequences used for siRNA construction.

Target genes	Target sequence (5'-3')
si-PTTG3P 1#	UGGCUACUCUGAUCUAUGUUGAUAA
si-PTTG3P 2#	GGGAGAUCUCAAGUUUCAAUAUCAU
si-ILF3	GCCATGTGATGGCAAAGCATT
si-MAP2K6	GGCTACTGATGGATTTGGA
si-E2F1	GACCACCUGAUGAAUAUCUTT
si-NC	UUCUCCGAACGUGUCACGUTT

Supplemental Table S4. A list of top 10 potential PTTG3P-interacting protein candidates in A549 cells based on RNA-protein pull-down assays and mass spectrometry analysis

Name	PepCount	UniquePepCount	CoverPercent	MW	PI
RRBP1	47	40	32.55%	152470.4	8.69
DHX9	44	36	30.63%	140956.9	6.41
NCL	77	35	37.75%	76613.47	4.6
LRPPRC	37	34	25.18%	157903.2	5.81
ILF3	50	29	43.87%	76049.9	7.62
NF90b	50	29	41.50%	76471.46	8.47
NF110b	48	29	33.41%	95776.73	8.9
HNRNPU	42	29	30.42%	90583.42	5.76
AHNAK	31	28	6.84%	629094.1	5.8
SPT16	30	26	28.75%	119912.4	5.5

Supplemental Table S5. Differential abundance of mRNAs ((Log2FoldChange >1.5) in si-PTTG3P A549 cells

Gene_name	Log2FoldChange	P value	Gene_biotype	Style
PTTG1	-5.143656756	1.13E-302	protein_coding	down
KRT4	-5.068237748	1.03E-05	protein_coding	down
ANKRD2	-4.05432062	3.08E-61	protein_coding	down
PRKCB	-3.915287226	0.002064236	protein_coding	down
KRTAP4-1	-3.213682448	0.000264156	protein_coding	down
HLA-DMB	-3.135806389	6.35E-06	protein_coding	down
MAP2K6	-3.051556427	7.92E-44	protein_coding	down
LBP	-3.034056933	0.009946683	protein_coding	down
SLC8A1	-2.981619665	1.90E-17	protein_coding	down
HSD17B12	-2.91786757	3.84E-164	protein_coding	down
KRT20	-2.738997033	0.00935964	protein_coding	down
CSTA	-2.656059346	0.003800728	protein_coding	down
MAD2L1	-2.609579929	1.09E-176	protein_coding	down
RORC	-2.607473595	0.016154773	protein_coding	down
H2AFV	-2.603629783	0	protein_coding	down
SPDEF	-2.601865833	1.72E-06	protein_coding	down
EFHC2	-2.570821853	0.017982421	protein_coding	down
CMTM6	-2.557342876	4.83E-156	protein_coding	down
TNFSF13B	-2.552349451	0.00457468	protein_coding	down
SELENOI	-2.550330451	0	protein_coding	down
PDE3A	-2.541068431	2.80E-12	protein_coding	down
TEX19	-2.536914651	0.022888767	protein_coding	down
FGF21	-2.534231339	0.000440022	protein_coding	down
NFU1	-2.523114625	1.49E-83	protein_coding	down
BCAS1	-2.511157647	1.91E-07	protein_coding	down
FCGBP	-2.478757157	9.19E-76	protein_coding	down
UGT2B11	-2.472424646	0.01522759	protein_coding	down
KIF5C	-2.448914879	1.01E-11	protein_coding	down
ANGPT1	-2.439557875	0.00786259	protein_coding	down
TNNC1	-2.431551755	0.000702829	protein_coding	down
MPLKIP	-2.404455696	6.23E-102	protein_coding	down
SNAPC1	-2.393187289	3.74E-80	protein_coding	down
RAB18	-2.385290303	2.99E-204	protein_coding	down
ANKRD1	-2.331477611	1.27E-93	protein_coding	down
AC136612.1	-2.300024179	0.013070163	protein_coding	down
CLDN1	-2.299588799	0	protein_coding	down
PRKAR2B	-2.277476766	3.19E-22	protein_coding	down
H3F3B	-2.275288699	0	protein_coding	down
ESCO2	-2.263049627	4.86E-72	protein_coding	down

MUC16	-2.240547662	1.24E-06	protein_coding	down
HS2ST1	-2.239453826	1.62E-116	protein_coding	down
PARP11	-2.227264726	1.83E-11	protein_coding	down
ANKRD30A	-2.216019237	0.00172565	protein_coding	down
SYT11	-2.21228937	8.09E-06	protein_coding	down
MROH2A	-2.194265547	0.00052964	protein_coding	down
YY2	-2.190923451	9.82E-08	protein_coding	down
POLR3G	-2.188611383	9.51E-58	protein_coding	down
TM4SF20	-2.179293189	1.62E-07	protein_coding	down
RASSF9	-2.174712097	2.78E-37	protein_coding	down
HR	-2.168309949	5.21E-134	protein_coding	down
GPR20	-2.166253865	0.000934147	protein_coding	down
CDK2AP1	-2.163729233	1.27E-99	protein_coding	down
FDXACB1	-2.148735235	0.001518501	protein_coding	down
IDI1	-2.147337873	5.32E-51	protein_coding	down
NRGN	-2.141937339	7.87E-26	protein_coding	down
VBP1	-2.140609786	1.03E-124	protein_coding	down
TMEM139	-2.092483663	3.03E-06	protein_coding	down
RNGTT	-2.08538049	1.37E-53	protein_coding	down
OXCT1	-2.077480948	3.00E-06	protein_coding	down
FAT3	-2.074696675	4.77E-14	protein_coding	down
ETV1	-2.069193103	2.61E-39	protein_coding	down
YIPF6	-2.061817003	6.11E-163	protein_coding	down
DENR	-2.015464192	3.26E-227	protein_coding	down
RADIL	-2.014431665	1.45E-38	protein_coding	down
NDC80	-2.01026825	1.83E-45	protein_coding	down
PLAC8	-2.009663346	0.001935627	protein_coding	down
MBNL1	-1.995170745	1.88E-247	protein_coding	down
STAC2	-1.992595924	0.004023766	protein_coding	down
NR1D2	-1.977755736	8.66E-159	protein_coding	down
NCOA7	-1.976778564	3.55E-194	protein_coding	down
HLF	-1.976355895	2.64E-08	protein_coding	down
CCDC3	-1.968357484	1.82E-06	protein_coding	down
SEMA3G	-1.964027137	7.54E-05	protein_coding	down
EIF5A2	-1.953986584	2.09E-38	protein_coding	down
MITF	-1.952171615	1.80E-120	protein_coding	down
NDC1	-1.949116922	4.86E-110	protein_coding	down
DEPDC1	-1.936106138	2.37E-83	protein_coding	down
HHIP	-1.932842081	4.81E-09	protein_coding	down
PTGES3	-1.932748666	0	protein_coding	down
MET	-1.929421497	0	protein_coding	down
C5orf22	-1.923411272	2.62E-62	protein_coding	down
SPAG16	-1.920998279	5.77E-35	protein_coding	down

MRAP2	-1.915719487	0.014646221	protein_coding	down
USP13	-1.912272042	1.35E-57	protein_coding	down
UHMK1	-1.911154948	4.35E-193	protein_coding	down
AK6	-1.904874385	7.21E-53	protein_coding	down
GUCY1A2	-1.900385835	2.88E-05	protein_coding	down
FUT1	-1.899316648	3.64E-24	protein_coding	down
PREPL	-1.89525283	8.36E-136	protein_coding	down
FSIP1	-1.893227698	0.000231793	protein_coding	down
DIRAS3	-1.886929226	7.44E-05	protein_coding	down
P2RX6	-1.876517598	1.18E-07	protein_coding	down
ADAM32	-1.875467701	5.18E-05	protein_coding	down
TERT	-1.874170278	5.36E-48	protein_coding	down
KBTBD6	-1.871286103	3.09E-30	protein_coding	down
YWHAH	-1.868027544	4.00E-183	protein_coding	down
CDC7	-1.867613998	1.88E-30	protein_coding	down
COQ2	-1.864798828	9.41E-63	protein_coding	down
ATP1A3	-1.861364906	1.69E-29	protein_coding	down
NEIL3	-1.855007818	3.98E-88	protein_coding	down
MYT1	-1.854521364	0.013204708	protein_coding	down
SYPL1	-1.852387143	1.86E-126	protein_coding	down
PMAIP1	-1.851018258	4.83E-124	protein_coding	down
USP53	-1.845785562	5.81E-166	protein_coding	down
MGAM	-1.844951452	2.26E-05	protein_coding	down
NDUFA6	-1.842899296	1.21E-101	protein_coding	down
DUSP19	-1.840215257	1.72E-06	protein_coding	down
CAMKV	-1.834492399	0.003269186	protein_coding	down
PM20D2	-1.834338008	4.04E-129	protein_coding	down
CCT8	-1.831791256	2.37E-252	protein_coding	down
AFMID	-1.826371308	2.64E-51	protein_coding	down
RRM2	-1.820091845	7.69E-90	protein_coding	down
ELOVL7	-1.806259897	2.90E-83	protein_coding	down
FAM72B	-1.806195761	2.11E-39	protein_coding	down
CXCL3	-1.803650187	7.49E-136	protein_coding	down
SERTAD4	-1.801472256	5.88E-10	protein_coding	down
MUC5B	-1.792015683	2.36E-17	protein_coding	down
SLC3A1	-1.789017386	4.24E-05	protein_coding	down
GSKIP	-1.775275194	1.88E-56	protein_coding	down
CACNA2D1	-1.762764594	4.41E-57	protein_coding	down
TPSG1	-1.762581374	0.008799695	protein_coding	down
NEK7	-1.760648463	6.08E-72	protein_coding	down
RBM7	-1.755960232	6.74E-21	protein_coding	down
ATP8A1	-1.754353441	0.005403259	protein_coding	down
SLFNL1	-1.748302943	2.97E-52	protein_coding	down

FAM72C	-1.74800644	9.02E-15	protein_coding	down
CYP4F3	-1.738698256	4.56E-50	protein_coding	down
TAF9B	-1.73162008	6.10E-52	protein_coding	down
RAB26	-1.728140174	6.01E-37	protein_coding	down
CTDSPL2	-1.728038268	4.10E-64	protein_coding	down
ATAD2	-1.727873521	1.43E-178	protein_coding	down
SUCNR1	-1.719194336	0.007132501	protein_coding	down
PTPN11	-1.71758286	4.08E-261	protein_coding	down
NT5DC1	-1.716718266	1.06E-44	protein_coding	down
EREG	-1.7133819	0	protein_coding	down
TM4SF4	-1.712120019	1.42E-15	protein_coding	down
KCNQ1	-1.709306754	0.000117194	protein_coding	down
SMIM20	-1.706950299	5.05E-26	protein_coding	down
LMBRD2	-1.706699151	2.34E-42	protein_coding	down
PDIA2	-1.703357773	0.010791887	protein_coding	down
SPOPL	-1.701298513	3.25E-34	protein_coding	down
FAM72A	-1.69783531	5.65E-24	protein_coding	down
STK24	-1.696966404	2.95E-105	protein_coding	down
ANP32E	-1.687835299	1.62E-180	protein_coding	down
PARP2	-1.681573469	2.09E-71	protein_coding	down
ISOC1	-1.674594461	2.30E-44	protein_coding	down
MAP2K6	-1.665948793	1.00E-18	protein_coding	down
PHF6	-1.662488197	2.69E-104	protein_coding	down
KCNK5	-1.653169195	6.31E-09	protein_coding	down
UTP15	-1.650740584	3.14E-52	protein_coding	down
EEF1AKMT2	-1.646651535	7.16E-12	protein_coding	down
VMA21	-1.645941575	9.02E-99	protein_coding	down
SAAL1	-1.641011861	5.79E-67	protein_coding	down
CXCL5	-1.639649284	0	protein_coding	down
CHRNA5	-1.639320328	3.69E-26	protein_coding	down
RAB30	-1.637000147	7.30E-20	protein_coding	down
HTATSF1	-1.635145105	1.63E-87	protein_coding	down
SDE2	-1.621021515	9.12E-79	protein_coding	down
INPP4B	-1.620728572	1.05E-35	protein_coding	down
CLSPN	-1.616493513	2.50E-50	protein_coding	down
IQGAP2	-1.616184975	2.97E-20	protein_coding	down
UBXN2B	-1.612028243	1.76E-58	protein_coding	down
NTRK3	-1.611302516	6.32E-78	protein_coding	down
ANKRD46	-1.605821526	1.54E-17	protein_coding	down
ZFR	-1.604445092	5.16E-154	protein_coding	down
GOLGA4	-1.603681016	1.71E-158	protein_coding	down
C4orf46	-1.600024591	1.21E-40	protein_coding	down
ALDH3A1	-1.594376867	0	protein_coding	down

ATP2B1	-1.59432475	9.76E-216	protein_coding	down
LMO1	-1.593495198	0.000130883	protein_coding	down
RAD54B	-1.590053748	1.16E-11	protein_coding	down
CCDC112	-1.589565687	9.44E-29	protein_coding	down
LGI3	-1.587296869	8.41E-15	protein_coding	down
ASAH2B	-1.585729815	1.23E-13	protein_coding	down
DAW1	-1.583387949	2.37E-07	protein_coding	down
MMGT1	-1.580434016	5.79E-45	protein_coding	down
RNF150	-1.579868146	0.002077133	protein_coding	down
MOSPD3	-1.5764689	1.68E-37	protein_coding	down
RAB27B	-1.570384736	1.04E-131	protein_coding	down
ALDH1A1	-1.568016909	0	protein_coding	down
WDR75	-1.56718908	6.46E-131	protein_coding	down
RNF219	-1.565934826	2.33E-15	protein_coding	down
C10orf88	-1.557577152	1.36E-36	protein_coding	down
HTRA3	-1.554398933	1.73E-05	protein_coding	down
NHLRC2	-1.550661203	7.26E-95	protein_coding	down
COQ7	-1.550090871	1.04E-37	protein_coding	down
PPAT	-1.549239074	1.81E-72	protein_coding	down
ABLIM2	-1.547974958	0.001010141	protein_coding	down
SLC7A2	-1.54574793	1.25E-39	protein_coding	down
CD2AP	-1.545443099	1.89E-113	protein_coding	down
SSX2IP	-1.53934772	5.32E-39	protein_coding	down
ANK3	-1.538778547	0.001780861	protein_coding	down
KCNIP3	-1.538513479	0.007285501	protein_coding	down
NEK2	-1.537334135	3.93E-71	protein_coding	down
SLC39A10	-1.535458103	6.42E-54	protein_coding	down
GNB4	-1.533948651	2.16E-39	protein_coding	down
GAL3ST2	-1.532260147	0.011821569	protein_coding	down
USP33	-1.530972565	5.13E-74	protein_coding	down
MRPL13	-1.528057379	9.51E-53	protein_coding	down
SCD	-1.52626333	1.27E-252	protein_coding	down
SMAD6	-1.525264408	1.71E-161	protein_coding	down
RMI1	-1.523191703	2.26E-43	protein_coding	down
XPOT	-1.519261281	4.19E-249	protein_coding	down
CA13	-1.51832031	0.00656157	protein_coding	down
SLC7A11	-1.514653746	0	protein_coding	down
TMEM37	-1.514388527	7.40E-05	protein_coding	down
CYP4F11	-1.514070485	6.81E-112	protein_coding	down
RAB37	-1.513813776	5.53E-10	protein_coding	down
NEK4	-1.512773097	9.62E-40	protein_coding	down
FGG	-1.510920583	7.67E-05	protein_coding	down
PPP6R3	-1.510347801	1.95E-177	protein_coding	down

MZT2A	-1.50786614	2.59E-112	protein_coding	down
EEF1E1	-1.507560754	2.18E-16	protein_coding	down
FAM72D	-1.503852699	1.29E-23	protein_coding	down
ADSSL1	-1.502605002	1.73E-28	protein_coding	down
EPS8L3	-1.502391965	0.019668413	protein_coding	down
FGL1	-1.502369747	5.90E-08	protein_coding	down
NRAS	-1.501287479	6.04E-88	protein_coding	down
DCDC2C	5.420184	3.28E-05	protein_coding	up
TREM1	4.88522	0.000319565	protein_coding	up
LCP1	4.787601	7.03E-40	protein_coding	up
ALOX15	4.582328	0.001059991	protein_coding	up
PRSS22	4.154726	6.85E-34	protein_coding	up
GPR87	3.853125	7.57E-08	protein_coding	up
CNTFR	3.840772	0.00196189	protein_coding	up
GFRA2	3.59205	1.18E-18	protein_coding	up
NTSR1	3.526288	2.20E-62	protein_coding	up
CCL22	3.500083	2.22E-19	protein_coding	up
WNT7A	3.458637	0.000179216	protein_coding	up
ZP4	3.368856	0.012451262	protein_coding	up
IL36B	3.215263	0.00059773	protein_coding	up
AQP9	3.085714	0.001438405	protein_coding	up
IL6R	3.021747	1.26E-268	protein_coding	up
MARVELD3	2.999119	4.68E-19	protein_coding	up
SEMA7A	2.941403	8.52E-86	protein_coding	up
STC1	2.905013	6.55E-69	protein_coding	up
SHISA9	2.878555	1.24E-06	protein_coding	up
PHF24	2.86691	0.003764707	protein_coding	up
TFF1	2.863764	9.73E-18	protein_coding	up
MMP1	2.856737	0.000124758	protein_coding	up
HAS1	2.78831	0.00532652	protein_coding	up
INHBA	2.750481	4.32E-96	protein_coding	up
CHRDL2	2.680028	0.000100023	protein_coding	up
SYT5	2.677258	2.50E-10	protein_coding	up
OVOL2	2.639179	3.19E-06	protein_coding	up
RNF225	2.6199	0.004756762	protein_coding	up
CORO2B	2.608487	3.05E-19	protein_coding	up
TMEM61	2.5889	0.000221179	protein_coding	up
EPPK1	2.588441	2.29E-08	protein_coding	up
SLCO1A2	2.554811	0.018032073	protein_coding	up
FFAR1	2.509693	0.016996876	protein_coding	up
RSAD2	2.508455	2.01E-08	protein_coding	up
COL5A3	2.478773	3.98E-09	protein_coding	up
SCARF1	2.448163	3.08E-12	protein_coding	up

C2orf15	2.435638	1.31E-06	protein_coding	up
KRT6B	2.403636	0.012262866	protein_coding	up
MMP9	2.39019	1.99E-05	protein_coding	up
ANKRD22	2.387084	5.35E-08	protein_coding	up
SOWAHB	2.381208	0.003344322	protein_coding	up
IGFBP5	2.368767	0.003331269	protein_coding	up
CEMIP	2.315757	1.45E-58	protein_coding	up
IFI44L	2.31196	1.38E-39	protein_coding	up
LAMP3	2.310582	6.23E-41	protein_coding	up
GYS2	2.304271	0.008559589	protein_coding	up
SLC16A11	2.294329	1.70E-08	protein_coding	up
MAF	2.285701	1.71E-08	protein_coding	up
HSH2D	2.277163	2.68E-10	protein_coding	up
LUM	2.267762	3.93E-06	protein_coding	up
RASL10A	2.265148	0.001327697	protein_coding	up
CDH3	2.261958	1.06E-13	protein_coding	up
HCAR3	2.241709	0.000223152	protein_coding	up
LIPG	2.213475	1.51E-09	protein_coding	up
ASB2	2.200121	8.72E-11	protein_coding	up
NLRC5	2.198508	1.30E-123	protein_coding	up
BTG2	2.185678	6.42E-203	protein_coding	up
FHDC1	2.183378	4.60E-41	protein_coding	up
IL10RA	2.176225	0.006389727	protein_coding	up
IL1RN	2.167602	0.009695572	protein_coding	up
ARL14	2.165026	5.72E-07	protein_coding	up
LRRC66	2.16321	0.000253777	protein_coding	up
FAM83B	2.162709	0.001640752	protein_coding	up
KISS1	2.16001	0.009149537	protein_coding	up
CLEC2B	2.139635	2.04E-05	protein_coding	up
SYT9	2.136678	3.25E-06	protein_coding	up
SCN3B	2.12997	0.00024658	protein_coding	up
F11R	2.109067	4.14E-204	protein_coding	up
SLC9A3	2.104261	8.24E-44	protein_coding	up
IL1RAP	2.104042	1.60E-89	protein_coding	up
DISP2	2.100769	2.84E-13	protein_coding	up
SPINT1	2.100315	1.11E-19	protein_coding	up
LYPD3	2.088112	3.70E-15	protein_coding	up
FAM160A1	2.087997	9.02E-08	protein_coding	up
FGF11	2.084041	0.001114846	protein_coding	up
SNAI2	2.06168	5.05E-07	protein_coding	up
SLC38A7	2.057317	8.61E-162	protein_coding	up
GLIPR1	2.050965	1.38E-116	protein_coding	up
LAMC2	2.048803	0	protein_coding	up

IRF8	2.046633	0.001449076	protein_coding	up
SOX2	2.04114	2.41E-07	protein_coding	up
STK40	2.038071	2.15E-258	protein_coding	up
C7orf61	2.034318	0.001142644	protein_coding	up
SOWAHA	2.031508	0.011291714	protein_coding	up
IFI27	2.030437	5.41E-13	protein_coding	up
FAM156A	2.020527	0.000238948	protein_coding	up
GPR68	2.016876	1.63E-05	protein_coding	up
KCNE5	2.011066	0.008070202	protein_coding	up
C6orf58	2.007528	2.47E-07	protein_coding	up
RNF223	2.005912	3.62E-27	protein_coding	up
IRX4	1.99536	0.00021855	protein_coding	up
ACSM4	1.993257	0.016207078	protein_coding	up
TMEM88	1.990184	1.57E-06	protein_coding	up
MAPK13	1.989218	1.28E-08	protein_coding	up
ISM2	1.983207	2.62E-09	protein_coding	up
PTPRB	1.97713	8.99E-05	protein_coding	up
RASSF10	1.968884	6.83E-144	protein_coding	up
FAAH2	1.966373	0.008323351	protein_coding	up
TXNIP	1.964492	1.19E-27	protein_coding	up
TLR4	1.956177	0.009655051	protein_coding	up
AMDHD2	1.940597	1.87E-86	protein_coding	up
OLAH	1.935297	0.002391386	protein_coding	up
EPSTI1	1.932083	0.000524245	protein_coding	up
HPX	1.929047	0.000194309	protein_coding	up
SLAMF7	1.929027	7.70E-06	protein_coding	up
DCHS1	1.926478	0.002768089	protein_coding	up
NKX2-1	1.920858	0.020543562	protein_coding	up
CASP1	1.90025	0.003358104	protein_coding	up
MRGPRX3	1.893213	0.005572767	protein_coding	up
ALOXE3	1.879029	3.87E-55	protein_coding	up
BEST4	1.876736	0.010262511	protein_coding	up
TMC8	1.869933	1.76E-08	protein_coding	up
ITGA5	1.865178	0	protein_coding	up
EPCAM	1.86495	3.14E-14	protein_coding	up
CD163L1	1.863811	4.40E-14	protein_coding	up
C17orf107	1.86114	0.000216218	protein_coding	up
NECTIN4	1.856703	3.55E-07	protein_coding	up
SPOCD1	1.85576	4.07E-51	protein_coding	up
PAG1	1.847195	0.000355452	protein_coding	up
HIC2	1.822709	2.31E-104	protein_coding	up
GOLGA8K	1.82054	4.83E-06	protein_coding	up
ANTXR2	1.817563	2.49E-44	protein_coding	up

COL1A1	1.817428	6.46E-86	protein_coding	up
TRIM29	1.816964	3.48E-06	protein_coding	up
ITGA10	1.815326	5.39E-06	protein_coding	up
CLEC4E	1.81089	3.07E-08	protein_coding	up
CDS1	1.798317	7.75E-22	protein_coding	up
VAMP5	1.790842	0.019360447	protein_coding	up
ARHGDIB	1.784217	0.000149246	protein_coding	up
DUSP2	1.778348	0.001866041	protein_coding	up
SLC9C1	1.777764	0.007921002	protein_coding	up
KIAA1161	1.775748	1.16E-82	protein_coding	up
CRB3	1.772044	1.17E-24	protein_coding	up
PTAFR	1.765832	1.02E-33	protein_coding	up
CPLX1	1.76399	1.73E-10	protein_coding	up
ISG15	1.76262	6.93E-14	protein_coding	up
AP3B2	1.753331	0.000230933	protein_coding	up
RUNDC3A	1.747284	3.35E-07	protein_coding	up
ACHE	1.742017	1.69E-30	protein_coding	up
IFI44	1.73786	1.27E-31	protein_coding	up
SCX	1.736889	7.63E-23	protein_coding	up
KLC3	1.733035	6.16E-14	protein_coding	up
GPR157	1.729338	9.10E-97	protein_coding	up
RHCG	1.719683	3.60E-104	protein_coding	up
MDFI	1.719282	0.001495571	protein_coding	up
NPR1	1.716076	1.55E-20	protein_coding	up
GOLGA8R	1.710964	0.005582588	protein_coding	up
KIAA1683	1.709901	5.32E-18	protein_coding	up
DNAJA4	1.697241	0.000390417	protein_coding	up
IL7R	1.695475	0.004607314	protein_coding	up
ZNF165	1.689606	0.000218297	protein_coding	up
SUSD4	1.682734	0.012111721	protein_coding	up
LSR	1.681344	9.68E-89	protein_coding	up
SERPINB7	1.680901	8.48E-19	protein_coding	up
FOXD4	1.677194	1.73E-05	protein_coding	up
VGF	1.675271	1.20E-70	protein_coding	up
CFAP70	1.675269	9.69E-05	protein_coding	up
DCLK1	1.673443	1.35E-28	protein_coding	up
BSPRY	1.671962	0.017577728	protein_coding	up
GPR4	1.667762	0.017976291	protein_coding	up
FBXO10	1.667695	1.32E-27	protein_coding	up
AC025594.2	1.66464	2.97E-05	protein_coding	up
ARID3A	1.660646	5.55E-181	protein_coding	up
SLC15A3	1.657459	2.16E-06	protein_coding	up
OAS2	1.656068	7.83E-06	protein_coding	up

FAM102A	1.654051	4.80E-280	protein_coding	up
ECM1	1.652064	9.00E-17	protein_coding	up
TP53INP2	1.650204	2.62E-45	protein_coding	up
NPHS1	1.645997	1.13E-70	protein_coding	up
ATG9B	1.645015	6.21E-05	protein_coding	up
HCAR2	1.642721	0.00033775	protein_coding	up
EXOC3L2	1.641371	6.34E-32	protein_coding	up
ZFYVE26	1.640533	1.62E-123	protein_coding	up
PTPRR	1.637823	0.000819832	protein_coding	up
ENTPD2	1.636644	7.25E-06	protein_coding	up
SEMA4B	1.634114	2.16E-51	protein_coding	up
CHST2	1.62032	1.04E-16	protein_coding	up
ARVCF	1.611416	4.83E-37	protein_coding	up
SERPINA1	1.609421	0.003141818	protein_coding	up
PLA2G15	1.603787	8.12E-62	protein_coding	up
ARID3B	1.603734	1.32E-89	protein_coding	up
C11orf96	1.603256	3.92E-08	protein_coding	up
NPIPA5	1.602484	0.020844071	protein_coding	up
TMEM178A	1.601656	0.000467112	protein_coding	up
PPP1R3B	1.600693	2.34E-101	protein_coding	up
CCDC154	1.598524	0.00011445	protein_coding	up
DSC2	1.595966	3.55E-30	protein_coding	up
BMF	1.595505	7.58E-21	protein_coding	up
TPPP	1.595014	5.25E-48	protein_coding	up
SLC25A34	1.593424	0.00660146	protein_coding	up
GBP4	1.587856	4.16E-06	protein_coding	up
KCNAB3	1.585502	3.14E-21	protein_coding	up
IFFO1	1.583592	0.000245956	protein_coding	up
SCEL	1.583359	3.39E-06	protein_coding	up
BSN	1.577261	7.16E-13	protein_coding	up
MAPK8IP2	1.576977	1.95E-47	protein_coding	up
WDR81	1.574859	7.49E-163	protein_coding	up
NDRG1	1.574767	2.87E-10	protein_coding	up
UAP1L1	1.57418	3.08E-154	protein_coding	up
ADAM19	1.574173	2.53E-90	protein_coding	up
CXCR4	1.574173	0.003158906	protein_coding	up
WNT9A	1.573998	6.83E-59	protein_coding	up
TRIM22	1.57371	1.07E-10	protein_coding	up
ARHGEF5	1.571726	0.0001719	protein_coding	up
CRY2	1.571181	4.12E-47	protein_coding	up
ATP6V0D1	1.570906	3.12E-123	protein_coding	up
XAF1	1.568717	3.34E-05	protein_coding	up
GOLGA7B	1.568165	5.67E-47	protein_coding	up

ITGA2	1.568085	3.31E-266	protein_coding	up
MICAL2	1.564873	3.24E-295	protein_coding	up
MXD1	1.5647	1.70E-40	protein_coding	up
JARID2	1.561582	8.37E-106	protein_coding	up
LAMB3	1.560775	8.41E-202	protein_coding	up
ZFP92	1.558954	2.91E-07	protein_coding	up
TCAF2	1.558655	0.00757887	protein_coding	up
KMO	1.557976	0.016117254	protein_coding	up
IL24	1.553144	3.03E-06	protein_coding	up
SPNS2	1.553038	9.30E-22	protein_coding	up
VNN1	1.551602	0.000340127	protein_coding	up
PLXNA3	1.549667	1.91E-108	protein_coding	up
FUT4	1.545675	3.51E-32	protein_coding	up
SOX9	1.543255	5.79E-54	protein_coding	up
CCDC188	1.542645	0.003019616	protein_coding	up
CDC42BPG	1.540933	2.93E-33	protein_coding	up
STMN3	1.537856	2.41E-74	protein_coding	up
GDPD5	1.535463	2.88E-57	protein_coding	up
F3	1.53293	8.37E-21	protein_coding	up
NPTX1	1.527404	9.30E-110	protein_coding	up
CEACAM1	1.527171	1.09E-13	protein_coding	up
TYMP	1.520679	3.90E-134	protein_coding	up
SLC5A2	1.517471	0.009003227	protein_coding	up
HYAL1	1.516018	7.68E-11	protein_coding	up
LOX	1.513245	5.59E-10	protein_coding	up
TMEM106A	1.513193	7.57E-17	protein_coding	up
HES4	1.51162	1.63E-95	protein_coding	up
FAM228B	1.506485	5.22E-06	protein_coding	up
ACRBP	1.505354	0.00347861	protein_coding	up
PRDM8	1.504085	2.09E-07	protein_coding	up
